# Hyperspectral data-driven corn nitrogen monitoring: application and interpretability analysis of multi-source feature optimization and stacked ensemble learning methods

**DOI:** 10.3389/fpls.2026.1734394

**Published:** 2026-05-21

**Authors:** Haoquan Kong, Yingnan Gu, Pu Zhao, Yanyan Zheng, Li Tian, Qinghui Dong

**Affiliations:** 1 Institude of Agricultural Remote Sensing and Information, Heilongjiang Academy of Agricultural Science, Harbin, China; 2 College of Information and Electrical Engineering, Heilongjiang Bayi Agricultural University, Da, Qing, China

**Keywords:** canopy nitrogen content, ensemble learning model, feature optimization, fractional-order derivative, hyperspectral data, model interpretability

## Abstract

**Introduction:**

Accurate monitoring of canopy nitrogen content is essential for sustainable nitrogen management, yield improvement, and environmental protection in industrial maize production. However, the high dimensionality of hyperspectral data and the limited accuracy and interpretability of existing models hinder practical applications.

**Methods:**

This study was conducted in Heilongjiang Province, China, using the maize cultivar Jinboshi. Genetic Algorithm (GA), Successive Projections Algorithm (SPA), and their hybrid strategy were compared for spectral band optimization. Sensitive vegetation indices were selected using multiple evaluation criteria, and a 0–2 order fractional-order derivative (FOD) method was applied to construct optimal two-dimensional (2D) and three-dimensional (3D) spectral indices. A stacked ensemble learning model was developed using XGBoost, GBDT, and Ridge as base learners and Bayesian Ridge as the meta-learner. Interpretability techniques were applied to analyze feature contributions.

**Results:**

The GA–SPA hybrid strategy effectively improved key spectral band selection. The 3D spectral index based on FOD achieved superior performance compared to vegetation indices and 2D indices (R^2^p = 0.801, RMSEP = 0.481). The optimized multi-source feature set combined with the stacked ensemble model yielded the best performance (R^2^p = 0.826, RMSEP = 0.450). Features from the red-edge and near-infrared regions, along with the 3D index, were the primary contributors to model predictions, consistent with plant nitrogen physiology.

**Discussion:**

The proposed framework, integrating feature optimization, advanced modeling, and interpretability analysis, provides an effective tool for precise nitrogen management in industrial maize and supports improved production efficiency with reduced environmental impact.

## 1 Introduction

Corn is a globally important energy crop. It plays an irreplaceable role in the production of bioethanol, bioplastics, and other industrial chemicals (Shi et al., 2023). Nitrogen is a key element for corn growth and development. It directly affects photosynthesis, biomass accumulation, and the synthesis of important substances such as amino acids, proteins, and enzymes. Consequently, nitrogen has a profound impact on corn’s overall potential as an energy crop (Wang et al., 2017). Research has shown that increased nitrogen content in corn significantly promotes biomass accumulation and enhances ethanol production efficiency during the bioenergy conversion process (Karyoti et al., 2021).

Furthermore, appropriate nitrogen levels improve corn’s performance in biodiesel and biogas production. Nitrogen also enhances the material properties of corn, such as increasing fiber strength and durability. This, in turn, optimizes the quality of biodegradable plastics and composite materials (Kuglarz et al., 2020; Okab and Abed, 2022). However, excessive fertilization remains a widespread issue in modern agricultural production. It not only increases production costs but also triggers environmental problems such as water eutrophication and greenhouse gas emissions. Therefore, developing precise nitrogen management strategies is crucial. Balancing economic benefits with ecological sustainability in corn production has become a key pathway for promoting the efficient and green development of maize. However, achieving precise nitrogen management in maize is highly dependent on the ability to accurately monitor crop nitrogen status in a timely and non-destructive manner. Traditional nitrogen detection methods, such as the Kjeldahl method and dry combustion, are often labor-intensive, time-consuming, and destructive, making them unsuitable for large-scale and real-time field applications. Therefore, there is an urgent need for advanced monitoring technologies that can provide rapid, non-invasive, and spatially continuous assessment of nitrogen status.

In this context, the prerequisite for achieving precise nitrogen management is the reliable, real-time, and non-destructive monitoring of crop nitrogen status. In recent years, hyperspectral remote sensing technology has gradually replaced traditional nitrogen detection methods, such as the Kjeldahl method and dry combustion method, by capturing subtle spectral responses in the crop canopy that are closely related to chlorophyll and nitrogen content (Qiao et al., 2024). Numerous studies have shown that the combination of hyperspectral data and machine learning (ML) models has been widely applied in nitrogen monitoring for various crops (Ruan et al., 2023; Sun et al., 2023; Yang et al., 2023; Rawal et al., 2024). However, the practical application of this technology is limited by the challenges posed by its high-dimensional data structure. Redundant data and noise interference from numerous spectral bands can lead to the “curse of dimensionality,” weakening the robustness and generalization capability of the models (Cao et al., 2021). Therefore, extracting the most discriminative features from the vast amount of spectral information has become a key step in advancing the practical application of this technology. Feature engineering is not only an important step in data preprocessing but also the foundation for building high-precision, transferable models.

In response to this issue, extensive research has been conducted on multi-level feature extraction and optimization. In terms of wavelength selection, data-driven methods such as Genetic Algorithm (GA) and Successive Projections Algorithm (SPA) have been widely used to select key bands and alleviate multicollinearity problems (Chen et al., 2022; Yang and Hu, 2024). Although these methods have made significant progress, single methods are limited by their dependence on specific bands and may fail to capture the potential complementarity between different bands comprehensively. Furthermore, over-reliance on a single feature selection method can lead to overfitting, limiting the model’s generalization ability in complex environments. Hybrid strategies that combine multiple feature selection methods have therefore been proposed. They aim to maximize information extraction while avoiding the biases and limitations of individual methods (Lin et al., 2023; Qin et al., 2025).

On the other hand, vegetation indices enhance target signals by combining specific bands. They are widely used in remote sensing research, especially in hyperspectral data processing for estimating crop nitrogen content (Ma et al., 2022). However, vegetation indices based on empirical band combinations often show poor adaptability across different environments, leading to a lack of universality among regions and conditions. Traditional vegetation indices also generally fail to fully account for differences in vegetation types or growth stages, further restricting their application range. To improve the efficiency of maize nitrogen content detection, selecting appropriate vegetation indices or combining multiple indices has become a key strategy to enhance model accuracy and robustness (Sellami et al., 2022) constructed vegetation indices based on prior experience to assess the physiological parameters of sweet corn. Through correlation analysis and multiple linear regression, they identified the optimal vegetation indices (such as CARI, DD, REIP, and Clred-edge) for monitoring yield and physiological responses under different water-nitrogen conditions, revealing the importance of the red-edge spectral region in identifying water-nitrogen stress.

In recent years, exploring deeper information within spectral data has become a breakthrough approach. Fractional-order differentiation (FOD), as a tool that extends integer-order calculus, has shown unique advantages in capturing subtle spectral variations and suppressing background noise (Li et al., 2023a). Compared to traditional differentiation methods, FOD can reveal more detailed spectral fluctuations, enhance useful signals, and effectively suppress noise, thereby improving the extraction of valuable information from hyperspectral data and significantly enhancing the accuracy of crop nitrogen content prediction (Yang et al., 2022) studied the impact of FOD on soil spectral reflectance and found that FOD enhances the correlation with soil total nitrogen (STN) content, especially within the order range of 0-1. As the order increases, the correlation gradually strengthens, providing an effective method for hyperspectral monitoring of soil STN. The two-dimensional and three-dimensional spectral indices constructed based on FOD can capture more complex interactions between bands and enhance the sensitivity and accuracy of nitrogen content monitoring through multi-dimensional feature fusion (Tang et al., 2025). In particular, the three-dimensional spectral index can extract more subtle differences related to nitrogen content across multiple spectral dimensions, providing a new approach for precise monitoring of crop nitrogen status. Despite significant progress, there is still a lack of an integrated research framework that systematically combines and compares multi-level feature optimization paths, ranging from band selection, vegetation index screening to high-dimensional spectral index construction. This gap remains, especially in the estimation of nitrogen content in key crops like maize.

To fully leverage the potential of feature optimization, advanced modeling methods must be integrated. Although machine learning models such as Support Vector Regression (SVR) and Gradient Boosting Decision Trees (GBDT) have demonstrated good performance in capturing the complex nonlinear relationships between spectra and nitrogen content, single models often face performance bottlenecks (Zhong et al., 2024). Stacked ensemble learning, which combines predictions from multiple heterogeneous base models through a meta-learner, can effectively enhance the model’s robustness and predictive accuracy (Xuan et al., 2025). While this strategy has achieved success in various fields, its application in industrial crop nitrogen management is still in its early stages. Moreover, the “black-box” nature of ensemble models may limit their acceptance in agricultural practices. Therefore, improving the interpretability of models has become a necessary condition for promoting their practical application. SHapley Additive exPlanations (SHAP) can quantify the contribution of each input feature to the model’s output, thereby transforming complex models into transparent tools that provide scientific insights (Ahmed et al., 2024).

**Figure 1:**
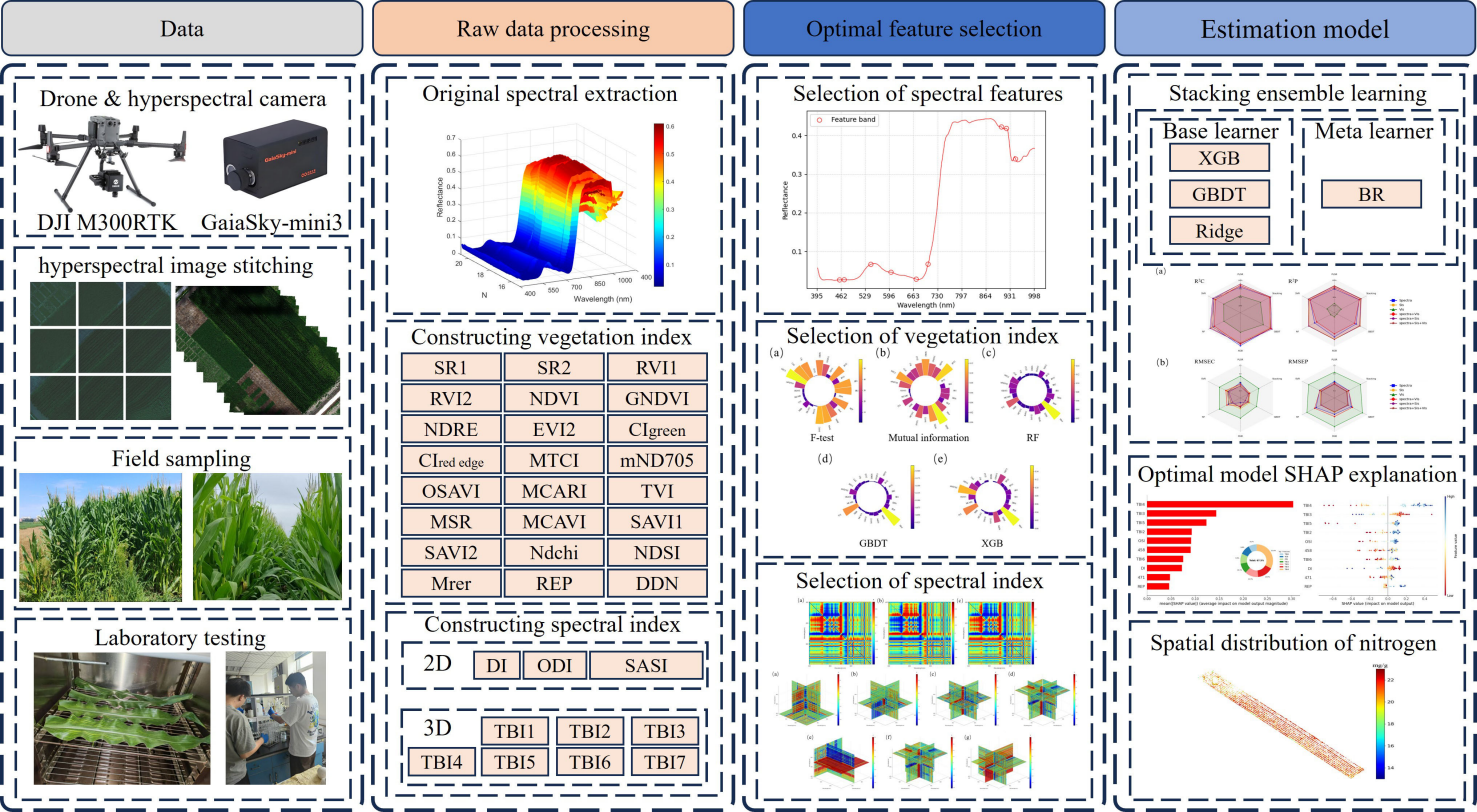
The overview of research technical route.

To address the aforementioned research gap, this study develops a systematic analytical framework aimed at achieving high-precision and interpretable estimation of nitrogen content in maize canopy (the technical approach is shown in **Figure 1**). The main contributions of this study are as follows: (1) A hybrid feature selection strategy combining GA and SPA is proposed and validated, and its performance in identifying key spectral bands is systematically evaluated; (2) New spectral features, particularly three-dimensional spectral indices, are constructed based on FOD, and compared with traditional vegetation indices and two-dimensional indices; (3) The synergistic effect of multi-source optimized feature fusion is explored, and its impact on model performance enhancement is assessed; (4) A stacked ensemble model is constructed and systematically compared with several individual models; (5) The SHAP interpretability framework is applied to analyze key spectral features and validate their consistency with plant physiological mechanisms. By systematically completing these tasks, this study not only provides a reliable method for precise nitrogen management in industrial maize but also aims to deepen the understanding of crop nitrogen status monitoring mechanisms through interpretable spectral analysis, offering scientific support for building an efficient and sustainable bio-based industrial chain.

## 2 Materials and methods

### 2.1 Experimental design

**Figure 2:**
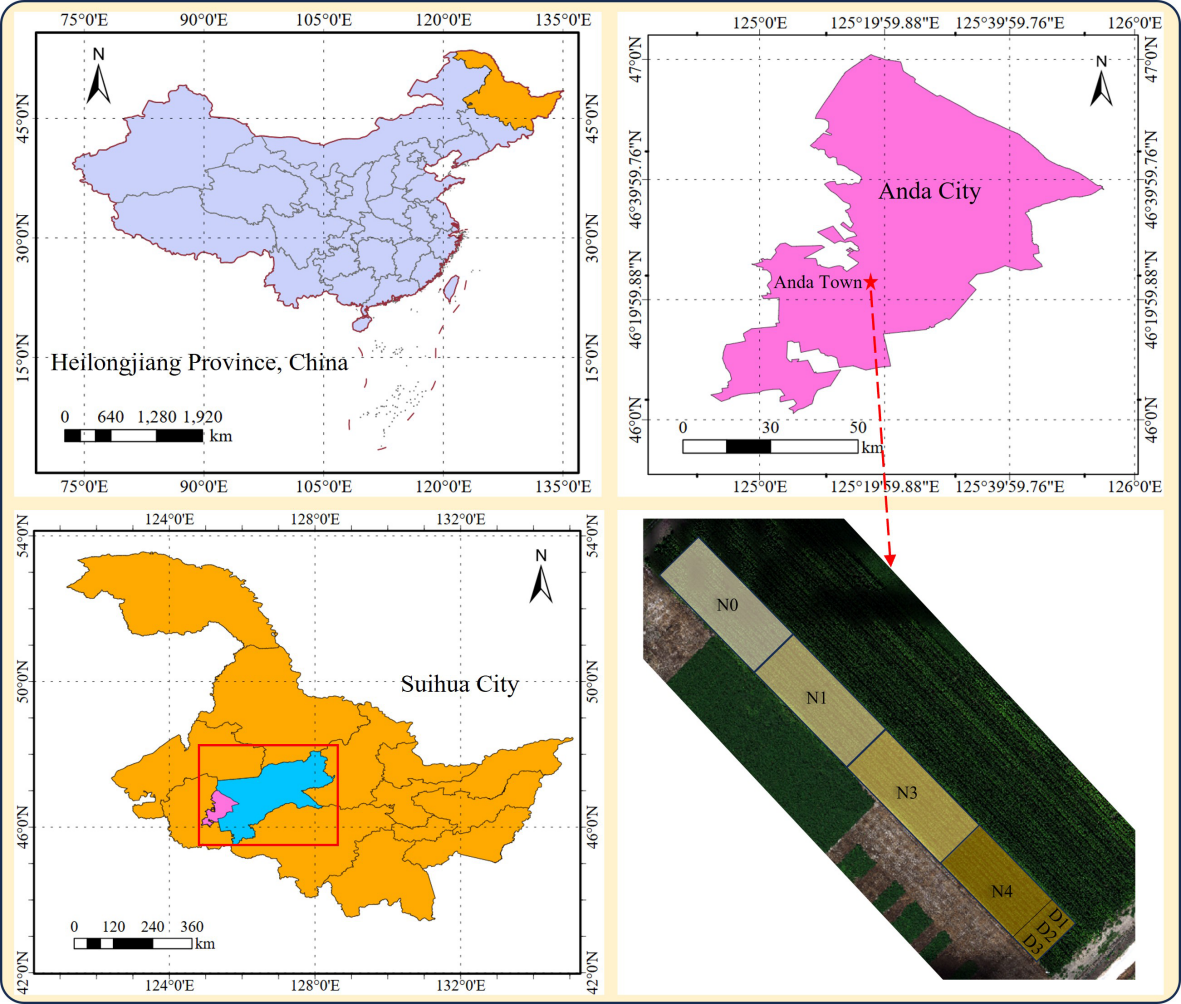
Location of study areas and experimental plots.

This study was conducted in 2024 at the Anda Agricultural Science and Technology Park in Daqing City, Heilongjiang Province, China (46°40′N, 125°35′E). The study area is flat and characterized by a temperate continental monsoon climate with abundant sunlight, providing favorable conditions for maize growth. The maize cultivar “Jinboshi 825” was sown in May 2024. Four nitrogen fertilizer treatments were established: N0 (0 kg/ha), N1 (125 kg/ha), N2 (225 kg/ha), and N3 (300 kg/ha). In addition, three planting density treatments were applied: D1 (5000 plants/acre), D2 (5500 plants/acre), and D3 (6500 plants/acre). Each treatment was arranged in a separate continuous plot, as shown in **Figure 2**. Within each treatment plot, multiple sampling points were uniformly distributed to collect both ground-measured and UAV-based hyperspectral data, ensuring sufficient sample representation for model development. Nitrogen fertilizer (urea, 46.4% N), phosphorus fertilizer (diammonium phosphate, 48% P), and potassium fertilizer (potassium chloride, 60% K) were applied. Base fertilizers were applied before the V1 stage, and additional nitrogen fertilizer was applied at the V6 and V12 stages. Data collection was conducted at the tasseling stage (VT stage) of maize, which is a critical period for nitrogen accumulation and canopy spectral response. All field measurements and UAV data acquisition were carried out under stable weather conditions (clear sky, wind speed < 3 m/s) to minimize environmental interference.

### 2.2 UAV data collection and preprocessing

In this study, the M300pro (DJI Technology Co., Ltd., Shenzhen, China) UAV equipped with the Gaiasky-mini3-VN hyperspectral imaging system (Sichuan Shuangli Hopu Technology Co., Ltd., China) was used for aerial data collection of maize. The detailed specifications are provided in **Supplementary Table 1**. To mitigate the effects of weather and solar azimuth on UAV imagery, aerial missions were planned between 10:00 AM and 1:00 PM, specifically under cloudless skies. This scheduling aimed to maintain uniform spectral illumination and minimize fluctuations induced by weather and sunlight. The flight operations and image acquisition were carried out using DJI GS PRO 2.0.17 (DJI Technology, China), with a flight altitude of 30 meters and image overlap set to 85% (forward) and 80% (side). Radiation calibration was performed before and after each flight using a standard radiometric calibration target. The acquired images were first atmospherically corrected using SpecView software to ensure reflectance consistency. Next, geometric correction was performed using HiRegistrator for precise spatial alignment and full-band registration. Finally, image stitching was carried out using Metashape, with image correction and alignment performed using ground control points (GCPs), generating high-precision orthophotos. Spectral normalization was then applied to maintain image consistency. The ENVI 5.3 software was used to define regions of interest (ROI) and extract the average reflectance for each band within each ROI.

### 2.3 Field data collection

Field nitrogen measurements and UAV image collection were conducted simultaneously on August 1st (tasseling stage). Fifty sampling points were randomly set within each fertilization plot, totaling 200 sampling points. At each sampling point, three representative upper canopy leaves (the 3rd fully expanded leaf from the top) were collected, as this leaf position shows stable morphology and strong representativeness in UAV imagery and is widely used for maize nitrogen status diagnosis (Ziadi et al., 2009). The collected leaves were placed in an oven, fixed at 105 °C for 30 min, and then dried to constant weight at 80 °C. After drying, samples were weighed, ground, and analyzed for nitrogen content using the Kjeldahl method.

### 2.4 Feature engineering

#### 2.4.1 Spectral band selection

The raw spectral data contains spectral information across the entire spectral range, but it may also include many redundant and irrelevant features, which could affect the calibration accuracy. Therefore, reducing redundant features and improving model accuracy becomes crucial. In this study, GA, SPA, and the GA-SPA combined algorithm were introduced to extract representative spectral features.

GA (Lin et al., 2024) is a global optimization algorithm based on the principles of natural selection and genetics, suitable for complex feature selection problems. In spectral feature selection, GA evaluates the quality of each band combination using a fitness function, prioritizing the retention of feature subsets with high fitness. It then generates new feature combinations through crossover and mutation, gradually approaching the optimal solution. This method effectively avoids getting trapped in local optima and demonstrates good adaptability in high-dimensional, nonlinear data. SPA (Wang et al., 2024) is a feature selection method based on stepwise projection, aimed at reducing multicollinearity in spectral data by selecting bands that are least correlated with each other. In each iteration, SPA calculates the projection differences of feature bands and selects the bands with the greatest projection differences, ensuring that the selected bands have minimal correlation, thus enhancing the model’s stability.

The GA-SPA method combines the global search capability of GA with the local selection ability of SPA, aiming to fully leverage the strengths of both. This method first uses GA to select potential important bands and then further optimizes the feature subset through SPA, retaining the bands with the highest information content and strongest correlation.

#### 2.4.2 Vegetation index selection

To identify vegetation indices (VIs) that are highly sensitive to maize nitrogen content, a multi-criteria feature selection strategy was employed. A total of 24 commonly used hyperspectral vegetation indices (**Table 1**) were initially considered (Li et al., 2017; Barbosa et al., 2021).

**Table 1:** Published hyperspectral VIs evaluated in this study.

Vegetation index	Equation	Reference
Simple ratio (SR) 1	R810/R560	(Xue et al., 2004)
SR 2	R750/R710	(Zarco-Tejada et al., 2002)
Ratio Vegetation Index (RVI)1	R800/R670	(Rouse, 1973)
RVI 2	R800/R670	(Floor et al., 1969)
Normalized Difference Vegetation Index (NDVI)	(R800−R680)/(R800+R680)	(Blackburn, 1998)
Green Normalized Difference Vegetation Index (GNDVI)	(R800−R550)/(R800+R550)	(Gitelson and Merzlyak, 1994)
Normalized Difference Red Edge Index (NDRE)	(R790−R720)/(R790+R720)	(Fitzgerald et al., 2006)
Enhanced Vegetation Index 2 (EVI 2)	2.5×(R800−R680)/(1+R800+ 2.4R680)	(Jiang et al., 2008)
Green Chlorophyll Index (CI_green_)	(R780/R550) − 1	(Gitelson et al., 2006)
Red Edge Chlorophyll Index (CI_red edge_)	(R780/R710) − 1	(Gitelson et al., 2006)
MERIS Terrestrial Chlorophyll Index (MTCI)	(R750−R710)/(R710−R680)	(Dash and Curran, 2007)
Modified Red-Edge Normalized Difference Vegetation Index (mND705)	(R750−R705)/(R750+R705− 2R445)	(Castro and Sanchez-Azofeifa, 2008)
Optimized Soil-adjusted Vegetation Index (OSAVI)	1.16×(R800−R670)/ (R800+R670 + 0.16)	(Rondeaux et al., 1996)
Modified Chlorophyll Absorption Ratio Index (MCARI)	1.2×[120×(R780−R550) − 200×(R800−R550)]	(Daughtry et al., 2000)
Transformed Vegetation Index (TVI)	0.5×[120×(R780−R550)−200×(R670−R550)]	(Gitelson et al., 1996)
Modified Simple Ratio (MSR)	(R800/R670−1)/sqrt (R800/R670 + 1)	(Chen, 1996)
Modified Chlorophyll Absorption Vegetation Index (MCAVI)	0.2×[2R800 + 1−sqrt(2R800 + 1)×2−8×(R800−R670)]	(Qi et al., 1994)
Soil Adjusted Vegetation Index (SAVI) 1	1.5×(R800−R670)/ (R80+R670 + 0.5)	(Huete, 1988)
SAVI 2	0.92×(R825−R735)/ (R825+R735−0.08)	(Huete, 1988)
Normalized Difference Chlorophyll (Ndchi)	(R925−R710)/(R925+R710)	(Lemaire et al., 2008)
Normalized Difference Spectral Index (NDSI)	(R788−R756)/(R788+R746)	(Li et al., 2013)
Modified Red Edge Ratio (Mrer)	(R759−1.8R419)/(R742−1.8R419)	(Feng et al., 2015)
Red Edge Position (REP)	700 + 40×[(R670+R780)/2−R700]/ (R740−R700)	(Guyot et al., 1988)
New Double Difference (DDN) Index	2R710−R660−R760	(Jin et al., 2020)

Specifically, Pearson and Spearman correlation analyses were first conducted to evaluate the linear and monotonic relationships between each vegetation index and nitrogen content, and indices with stronger correlations were preliminarily retained. Subsequently, an F-test was performed to assess the statistical significance of each index, while Mutual Information (MI) was used to quantify the nonlinear dependency between vegetation indices and nitrogen content. Furthermore, model-based feature importance analysis was carried out using Random Forest (RF), GBDT, and XGBoost to evaluate the contribution of each vegetation index to nitrogen prediction. To comprehensively integrate the results from multiple evaluation methods, an overall difference combination assessment (ODCA) approach was adopted. In this process, the ranking results obtained from different methods were first normalized to a unified scale, and a comprehensive score for each vegetation index was then calculated by aggregating its normalized rankings across all methods. Finally, the vegetation indices were ranked according to the comprehensive scores, and the top 10 indices were selected to construct the optimal vegetation feature set.

This multi-source evaluation framework enables the selection of robust and informative vegetation indices by jointly considering linear and nonlinear relationships as well as model-based contributions.

#### 2.4.3 Fractional-Order Differentiation and Spectral Index Construction

To enhance the sensitivity of spectral features to subtle biochemical variations, FOD was applied to the canopy spectral data. Compared with traditional integer-order derivatives, FOD enables continuous variation in derivative order, providing greater flexibility in capturing spectral characteristics. In this study, the fractional order ranged from 0 to 2 with an interval of 0.25, generating multiple transformed spectral datasets. Based on these FOD-transformed spectra, a series of two-dimensional (e.g., DI, OSI, SASI) and three-dimensional (e.g., TB1–TB7) spectral indices were constructed (**Table 2**). Subsequently, correlation analysis was performed between the constructed spectral indices and nitrogen content to identify the most sensitive band combinations. The indices with the highest correlation were selected to form the final spectral index dataset, thereby improving the accuracy and robustness of nitrogen estimation.

**Table 2:** The formulae of multi-band spectral indices used in this study.

Type	Spectral index	Formula	Reference
Two-band	DI	*R*_*λ*1_ −*R*_*λ*2_	(Jin et al., 2017)
	OSI	(1 + 0.45) (2*R*_*λ*2_ + 1)/(*R*_*λ*1_ + 0.45)	(Huang et al., 2024)
	SASI	(1 + 0.5) (*R*_*λ*1_ − *R*_*λ*2_)/(*R*_*λ*1_ + *R*_*λ*2_ + 0.5)	(Sharifi and Felegari, 2023)
Three-band	TBI_1_	*R*_*λ*1_/(*R*_*λ*2_*R*_*λ*3_)	(Wang et al., 2019)
	TBI_2_	*R*_*λ*1_/(*R*_*λ*2_ + *R*_*λ*3_)	(Wang et al., 2019)
	TBI_3_	(*R*_*λ*1_ − *R*_*λ*2_)/(*R*_*λ*1_ +*R*_*λ*3_)	(Wang et al., 2019)
	TBI_4_	(*R*_*λ*1_ − *R*_*λ*2_)/(*R*_*λ*1_ −*R*_*λ*3_)	(Lao et al., 2021)
	TBI_5_	(*R*_*λ*1_ + *R*_*λ*2_)/*R*_*λ*3_	(Lao et al., 2021)
	TBI_6_	(*R*_*λ*1_ − *R*_*λ*2_)/(*R*_*λ*1_ − *R*_*λ*2_) −(*R*_*λ*_1 −*R*_*λ*3_)	(Lao et al., 2021)
	TBI_7_	(*R*_*λ*1_ − *R*_*λ*2_) − (*R*_*λ*1_ −*R*_*λ*3_)	(Lao et al., 2021)

### 2.5 Nitrogen content prediction models

#### 2.5.1 Classical models

To comprehensively assess the contribution of different feature sets to maize nitrogen content prediction and enhance model performance, this study selected several classical regression models and ensemble learning methods. First, Partial Least Squares Regression (PLSR) was chosen to handle high-dimensional and highly correlated spectral data, as it effectively alleviates collinearity issues and extracts the most representative features (Cheng and Sun, 2017). SVR addresses nonlinear regression problems using kernel tricks, making it suitable for handling complex relationships in hyperspectral data (Zhong et al., 2024). RF reduces overfitting by integrating multiple decision trees, enhancing the model’s stability and robustness, particularly for high-dimensional feature processing (Chen et al., 2022). GBDT iteratively trains multiple decision trees to gradually reduce residuals, effectively fitting nonlinear relationships and complex interaction effects in the data (Li et al., 2024). Finally, XGBoost, as an improved version of gradient boosting trees, efficiently handles large-scale data and is especially suitable for complex regression tasks (Huang et al., 2022).

#### 2.5.2 Stacked ensemble learning models

**Figure 3:**
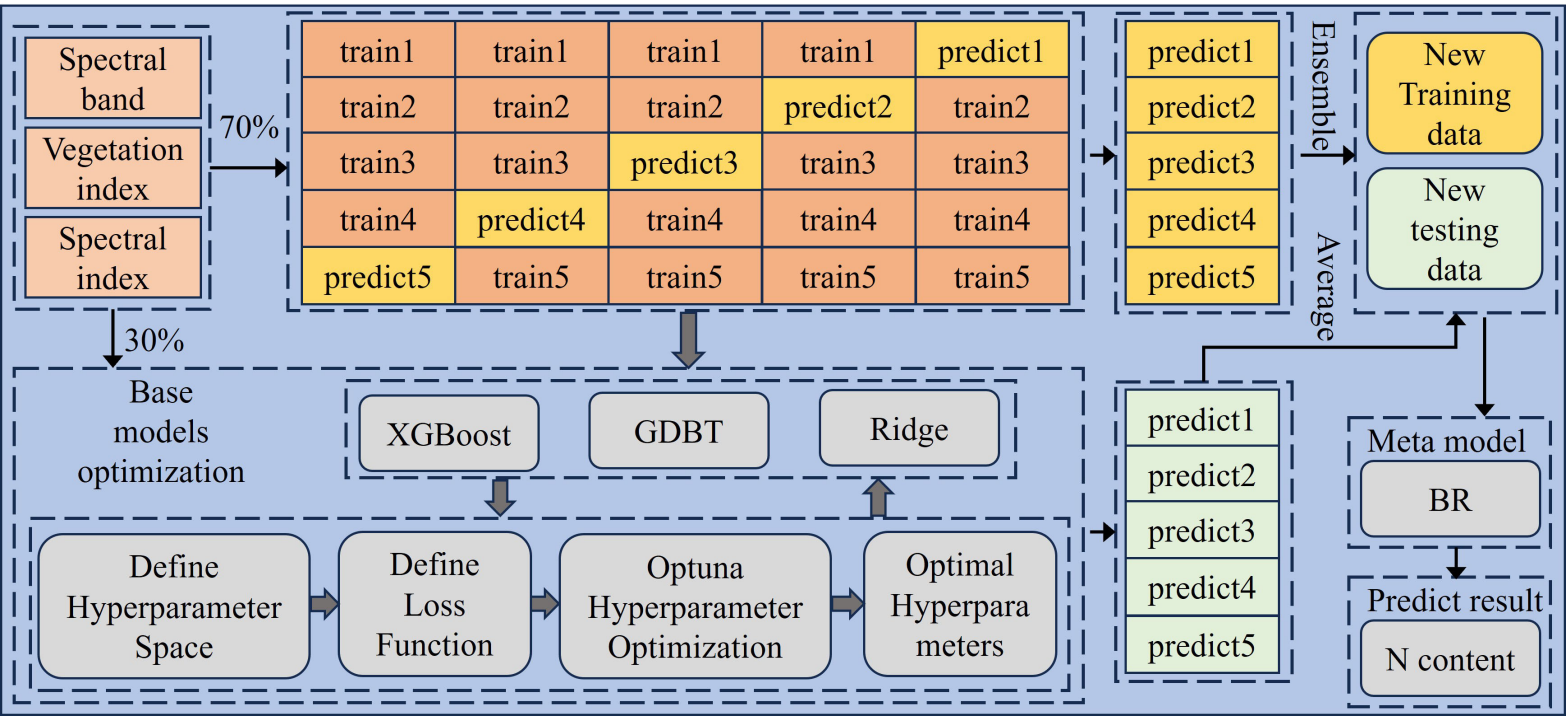
Schematic representation of stacking ensemble learning model architecture for prediction.

The stacked ensemble learning approach boosts prediction accuracy and stability by integrating the outputs of various base models, effectively addressing the shortcomings of individual models. To minimize overfitting and improve the model’s generalization capability, cross-validation techniques are utilized, ensuring the model’s reliability and robustness. In this study, we designed a stacked ensemble framework based on multiple base learners and a meta-learner (see **Figure 3**). The base learners include XGBoost, GBDT, and Ridge regression, while the meta-learner selects Bayesian Ridge regression.

XGBoost effectively handles high-dimensional data and captures complex nonlinear relationships through its regularization mechanism; GBDT, with its efficient modeling capability, is particularly suitable for noisy datasets; Ridge regression provides a stable linear foundation, compensating for the shortcomings of the previous two models when dealing with linear relationships (Ji et al., 2022). By combining the prediction results of these three base learners, the stacked model can simultaneously capture both nonlinear relationships and linear trends in the data, thereby providing more accurate and robust predictions. For the meta-learner, we chose Bayesian Ridge regression, which enhances model robustness by handling parameter uncertainty through Bayesian inference, making it particularly suitable for handling data with complex feature relationships and high levels of noise. Through this fusion, the stacked model not only improves prediction accuracy but also strengthens the ability to handle uncertainty in the data, further optimizing the nitrogen content prediction task in hyperspectral data analysis (Concepcion et al., 2025). Hyperparameter optimization is a critical step in model development. To achieve higher prediction accuracy and greater robustness, a grid search strategy was employed. Grid search identifies the optimal parameter combination by systematically traversing a predefined hyperparameter space. The detailed hyperparameter search ranges for each model are summarized in **Supplementary Table 2**. An overview of the training process is outlined as follows:

1. The complete dataset was randomly split into training (70%) and testing (30%) subsets to maintain consistency in data distribution.
2. Hyperparameters of the stacked model were optimized through grid search, which explores all possible hyperparameter combinations to select the optimal parameter set.
3. A five-fold cross-validation scheme was implemented, where the training set was divided into five equal subsets. Each subset was used as validation data once, with the remaining four subsets used for training the model.

### 2.6 Model performance evaluation

In this study, the performance of each model was evaluated using indicators such as the calibration coefficient of determination (R²c), calibration root means square error (RMSEC), prediction coefficient of determination (R²p), and prediction root mean square error (RMSEP). The larger the R²c and R²p, and the smaller the RMSEC and RMSEP, the better the model’s performance. The calculation formulas are as follows:


$$R^{2}=\frac{\sum_{i=1}^{n}{\left(y_{i}-{\bar{y}}_{i}\right)}^{2}-\sum_{i=1}^{n}{\left(y_{i}-{\hat{y}}_{i}\right)}^{2}}{\sum_{i=1}^{n}{\left(y_{i}-{\bar{y}}_{i}\right)}^{2}}$$

$$RMSE=\sqrt{\frac{\sum_{i=1}^{n}{\left(y_{i}-{\hat{y}}_{i}\right)}^{2}}{n}}$$

Here, $y_{i}$, ${\hat{y}}_{i}$ and ${\bar{y}}_{i}$ represent the test values of the response variable, the predicted values, and the mean values, respectively.

### 2.7 Model interpretation

To accurately interpret the nitrogen content prediction model, SHAP was applied to the model with the highest R² value. SHAP is a method that uses cooperative game theory principles to explain machine learning model outputs (Ahmed et al., 2024). In the context of machine learning, SHAP treats features as participants in a game, with the model output as the payoff. It quantifies the contribution of each feature to the model’s output by evaluating its impact on the prediction results, offering a clear explanation of the model’s decision-making process.

## 3 Results

### 3.1 Field nitrogen content analysis

**Supplementary Figure 1** presents the statistical results of maize canopy nitrogen content under different fertilization gradients. As shown in the figure, nitrogen content in maize significantly increased with the amount of fertilization (P< 0.05). The average nitrogen content for the N0 group was 17.5 mg/g, for the N1 group was 18.5 mg/g, for the N2 group was 19.5 mg/g, and for the N3 group was 20.0 mg/g. The standard deviation gradually increased, indicating greater variability in nitrogen content at higher fertilization gradients. These results suggest that fertilization gradients have a significant impact on the increase in maize nitrogen content.

### 3.2 Feature selection results

#### 3.2.1 Spectral band selection

**Figure 4:**
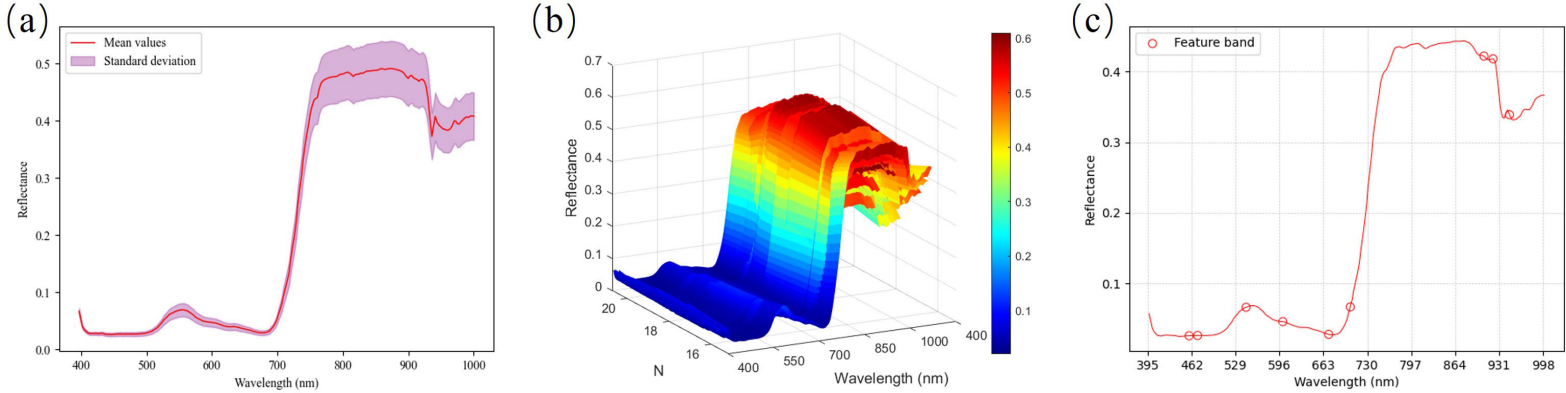
Original spectrum and feature optimization graph **(a)** Original spectral graph **(b)** Three-dimensional spectral profiles of samples with varying nitrogen contents (c) GA-SPA Band Selection Band Selection.

**Figure 4A** shows the original spectral curves of 200 maize canopy leaves extracted from the calibrated hyperspectral image ROIs. The spectral response of the leaves exhibits typical plant spectral features. High-nitrogen leaves have lower reflectance peaks in the 500–600 nm band, primarily due to strong absorption of green light, leading to reduced reflectance. In the 650–700 nm band, red light absorption is particularly intense, showing a distinct absorption feature. In the 900–950 nm band, the reflectance fluctuates, with a slight absorption peak, which is closely related to water absorption in plant leaves. The strong water absorption effect leads to a decrease in reflectance, indirectly affecting the measurement of nitrogen content. **Figure 4B** shows the relationship between spectral reflectance and nitrogen content, further indicating the sensitivity of different bands in reflecting differences in nitrogen content.

**Figure 5:**
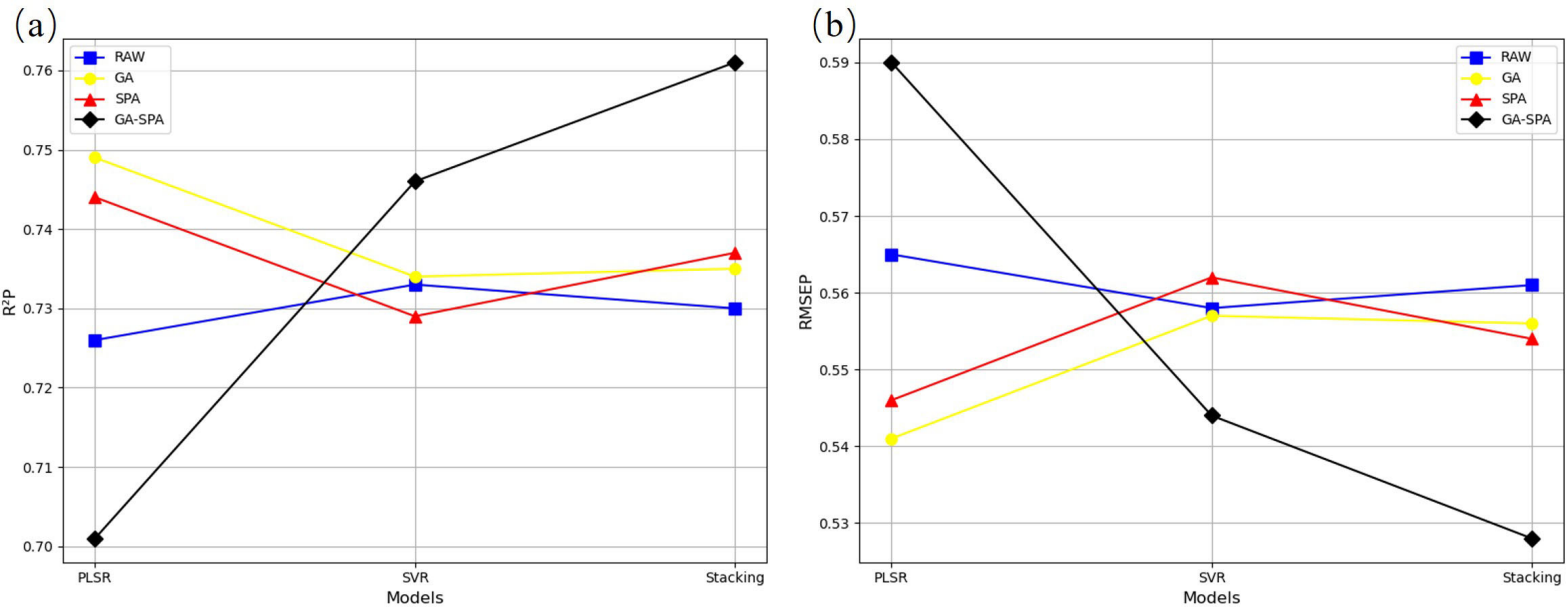
Accuracy statistics for N estimation with different feature selection **(a)** R2P **(b)** RMSEP

Due to the large amount of irrelevant information in the high-dimensional bands extracted, this study used GA, SPA, and GA-SPA methods (**Figure 4C**) for feature selection on the original spectral data, and evaluated the performance of these three methods in different machine learning models (PLSR, SVR, Stacking) (**Figure 5**; **Supplementary Table 3**). The results showed that the GA method performed stably across all models, especially showing good results in the PLSR and SVR models. The SPA method showed some improvement in PLSR but had limited performance in the SVR and Stacking models. The GA-SPA method performed best in the SVR and Stacking models, particularly in the Stacking model, where R²P reached 0.761, and RMSEP decreased to 0.528, significantly improving prediction accuracy. This further validates the effectiveness of combining GA and SPA. Although the GA-SPA method performed weaker in PLSR, its advantage in handling high-dimensional data remains significant and can effectively improve model accuracy.

#### 3.2.2 Vegetation index selection

**Figure 6:**
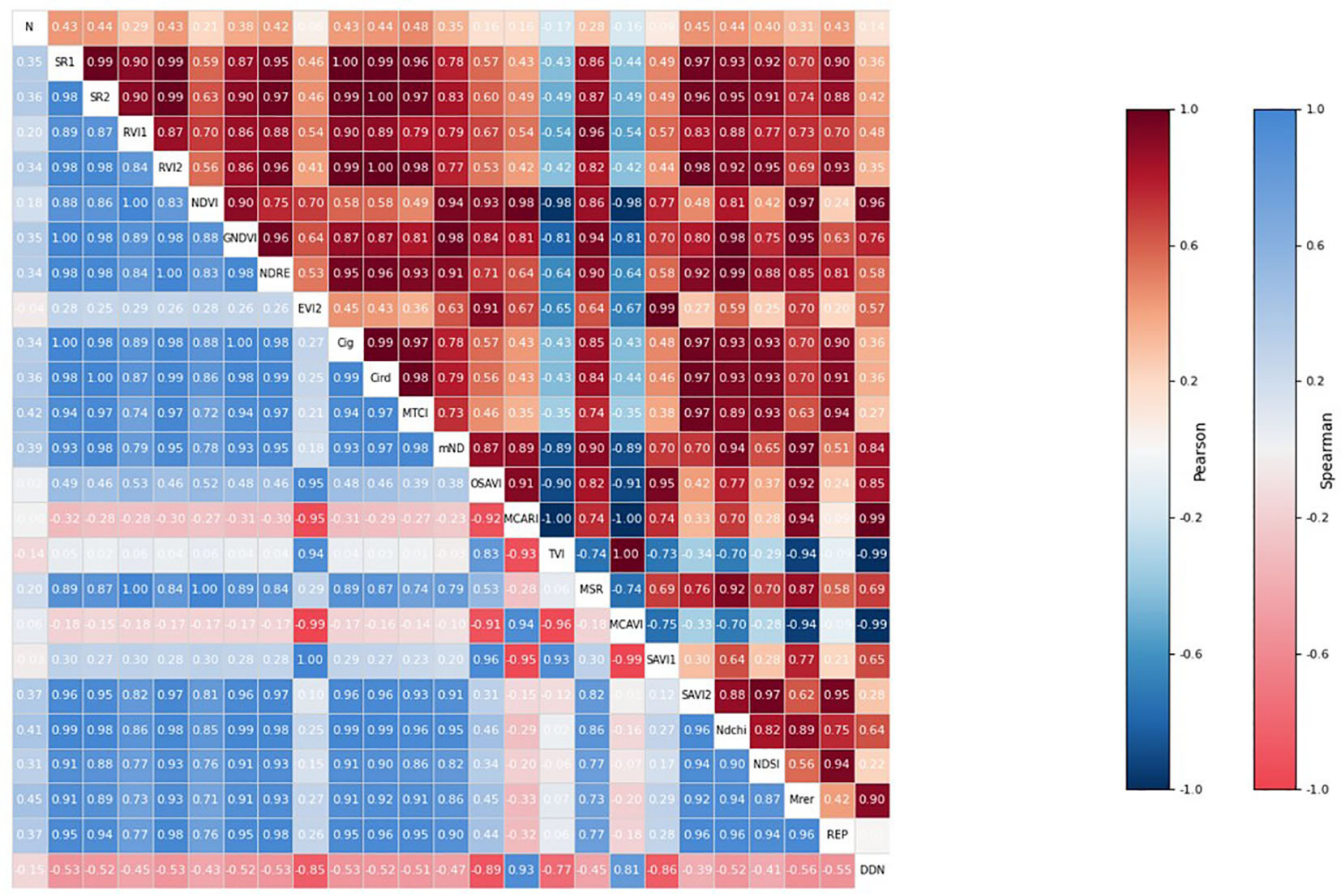
Pearson and Spearman correlation analysis diagrams of nitrogen content based on different vegetation indices.

Through Pearson and Spearman correlation analysis of vegetation indices (see **Figure 6**), the results indicate that the correlation between different vegetation indices and nitrogen content varies. In the Pearson correlation analysis, MTCI showed the strongest correlation (0.48), while TVI exhibited the weakest correlation (-0.17). In the Spearman correlation analysis, Mrer showed the strongest correlation (0.45), while DDN showed the weakest correlation (-0.15). Although the correlation between vegetation indices and nitrogen content is at a medium to low level, effective nitrogen content prediction is still achievable. However, some vegetation indices exhibited high correlations with each other (>0.95), indicating strong multicollinearity between them, leading to information overlap, which could potentially cause instability in the prediction results. Therefore, feature selection and optimization are necessary.

**Figure 7:**
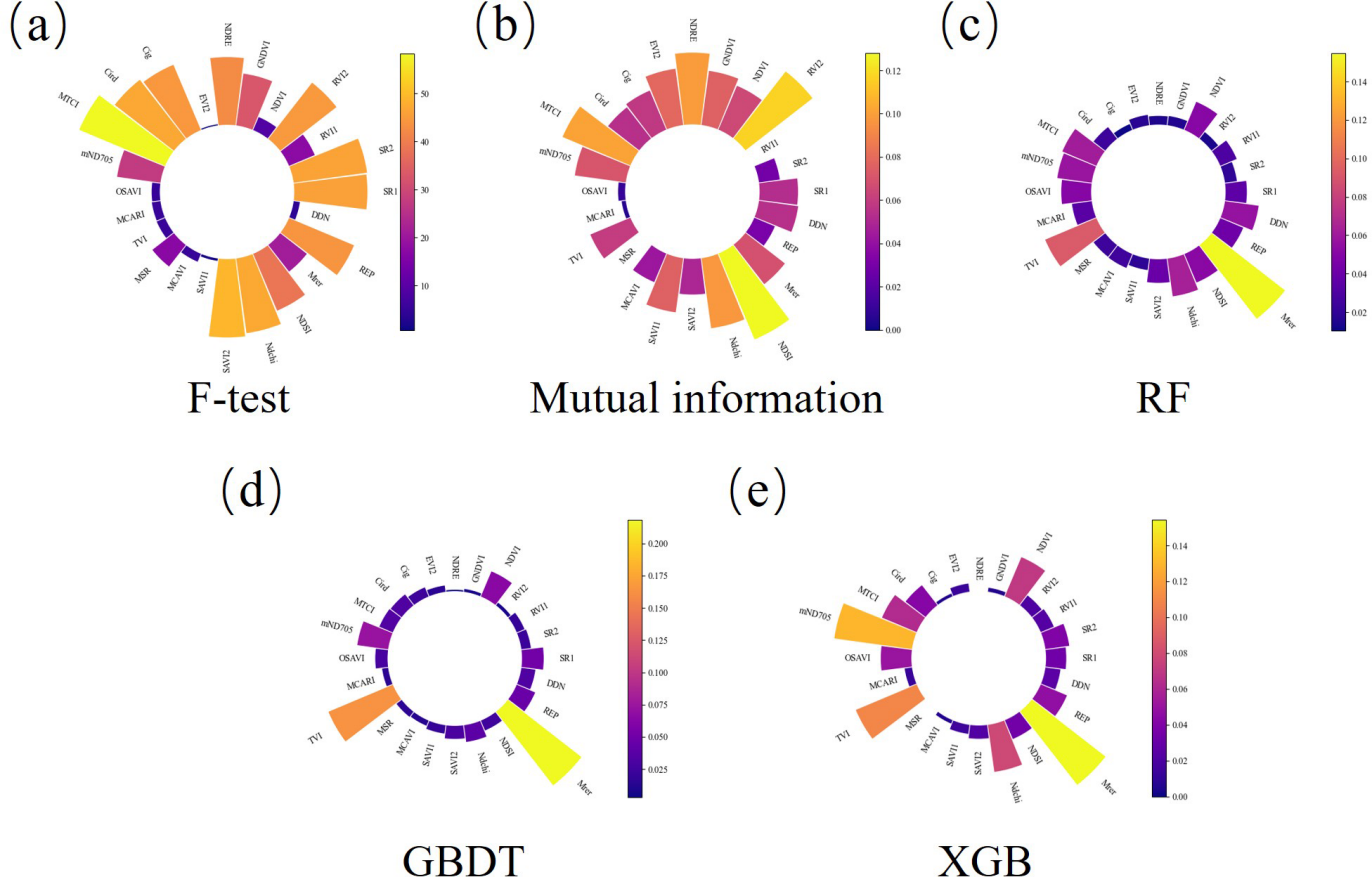
Ranking map of the contribution of vegetation indices based on different machine learning methods **(a)** F-test **(b)** Mutual information **(c)** RF **(d)** GBDT **(e)** XGB.

To further select important vegetation indices, F-test, Mutual Information, and algorithms such as RF, GBDT, and XGB were used to rank the different vegetation indices based on their impact on the nitrogen content prediction model. As shown in **Figure 7**, these vegetation indices produced slightly different rankings in the various methods. For example, MTCI showed a higher contribution in the F-test and mutual information evaluations, but a lower contribution in RF, GBDT, and XGB. To obtain more stable and high-contribution vegetation indices, the rankings from these seven methods were integrated using ODCA (**Table 3**). The top ten vegetation indices—MTCI, Ndchi, Mrer, mND705, SAVI2, CI_red edge_, SR1, REP, NDSI, and NDVI—provided the greatest contribution to the nitrogen content prediction model.

**Table 3:** Rank of importance of each vegetation index.

Components	Pearson	Spearman	F-test	Mutual information	RF	GBDT	XGB	Final rank (ODCA)
SR1	6	9	6	16	12	5	12	7
SR2	5	8	5	20	18	15	10	11
RVI1	15	15	15	24	14	17	14	19
RVI2	7	11	7	2	23	22	16	13
NDVI	17	17	17	11	8	4	5	10
GNDVI	12	10	12	8	21	23	20	16
NDRE	10	12	10	4	22	24	24	15
EVI2	22	22	24	6	20	19	18	23
CI_green_	8	13	8	13	24	14	22	14
CI_red edge_	4	7	4	15	16	10	9	6
MTCI	1	2	1	3	4	8	6	1
mND705	13	4	13	9	5	3	2	4
OSAVI	19	19	21	21	9	12	7	18
MCARI	18	20	19	22	13	20	19	22
TVI	24	23	18	12	2	2	3	12
MSR	16	16	16	23	17	18	23	21
MCAVI	23	18	20	18	15	21	21	24
SAVI1	21	21	23	7	19	16	17	20
SAVI2	2	5	2	17	11	11	15	5
Ndchi	3	3	3	5	3	7	4	2
NDSI	11	14	11	1	7	13	11	9
Mrer	14	1	14	10	1	1	1	3
REP	9	6	9	19	10	6	8	8
DDN	20	24	22	14	6	9	13	17

### 3.3 Feature construction results

#### 3.3.1 Fractional order differential preprocessing

**Figure 8:**
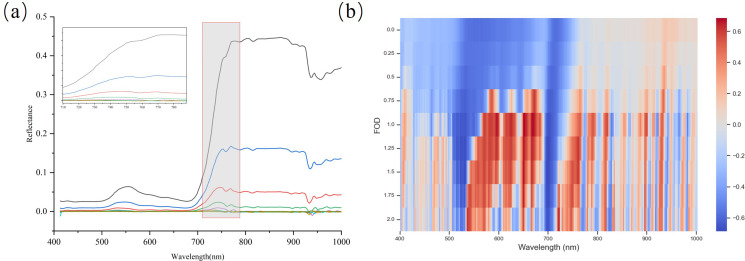
FOD processing of corn spectra **(a)** Spectral curves of various derivative orders, ranging from the original spectrum to 0.1–2nd order derivatives **(b)** Correlation coefficients between nitrogen content (N) and spectra with different derivative orders in the 400-1000 nm range.

**Figure 8A** shows the changes in the reflectance curves of maize canopy spectra before and after FOD transformation. As the fractional order increases from 0 to 2, the spectral reflectance intensity gradually decreases, leading to narrower spectral absorption regions and a reduction in the differences between the canopy spectra. In the original spectra, the reflectance is relatively low in the 400–700 nm range, sharply rises in the 700–800 nm range, and then flattens out, with a decline near 930 nm due to the water absorption peak. At fractional orders of 0.25-0.5, the spectral curves show a clear overall downward trend, enhancing the local inflection points of the spectra, making the slight fluctuations in reflectance caused by nitrogen content changes more significant. When the fractional order increases to 0.75-1.5, the spectral curve smooths, especially reducing the reflectance variations in the visible light range, indicating that baseline drift has been effectively eliminated while preserving the reflectance peaks and absorption valleys. Between orders of 1.75-2.0, the spectral reflectance gradually approaches zero. High-order derivatives amplify the high-frequency variations in the spectrum, causing what should be stable features to be over-amplified, which may exacerbate noise and increase the complexity of the analysis.

The nitrogen content correlation analysis was performed on the spectra after fractional order differentiation (see **Figure 8B**). The results show a trend of correlation first increasing and then decreasing. The original spectrum is negatively correlated with nitrogen content in the visible light region and has a low correlation in the near-infrared region. As the fractional order increases to 0.75, the correlation coefficients at the 570, 660, and 930 nm bands gradually increase, and a positive correlation peak appears. As the fractional order increases from 0.75 to 1.5, the correlation coefficient significantly increases in the 570–660 nm range, and more positive correlation peaks appear in the 720–820 nm range. The correlation coefficient reaches its maximum at the 644 nm band with a fractional order of 1.25 (R = 0.685). After the 1.75-2.0 fractional orders, the correlation coefficient gradually decreases, which may be due to the high-order differentiation amplifying some high-frequency fluctuations unrelated to nitrogen content, leading to a decrease in correlation.

#### 3.3.2 Optimal spectral index band combination and performance analysis

**Figure 9:**
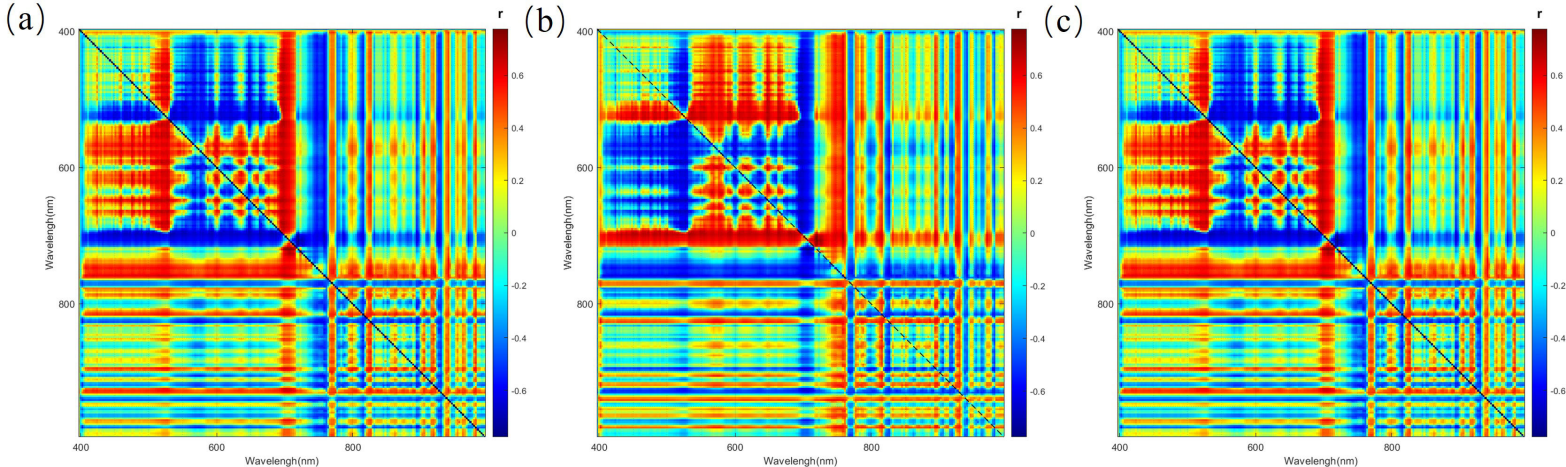
The Pearson correlation matrix of nitrogen content in corn and two-dimensional spectral indices **(a)** DI **(b)** OSI **(c)** SASI.

**Figure 10:**
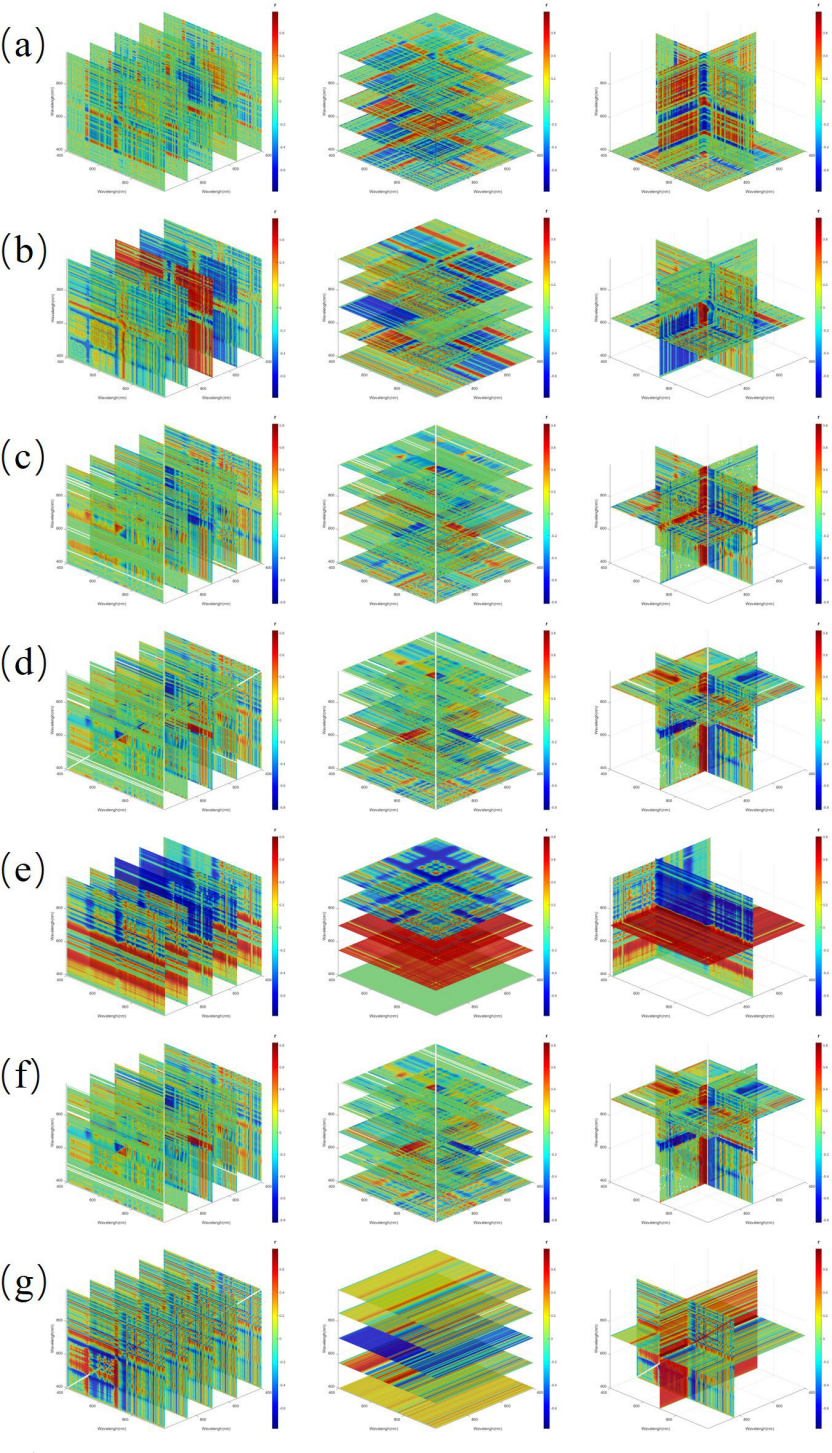
The Pearson correlation matrix of nitrogen content in corn and three-dimensional spectral indices **(a)** TBI1 **(b)** TBI2 **(c)** TBI3 **(d)** TBI4 **(e)** TBI5 **(f)** TBI6 **(g)** TBI7.

To identify spectral indices that are effective for nitrogen inversion within the high-dimensional features derived from the FOD method, a comprehensive search was performed to select the band combinations exhibiting the highest correlation with nitrogen content (see **Supplementary Table 4** and S5). In the two-band combinations, DI at the 1.5 order had the highest correlation, 0.778, OSI at the 1.5 order had the highest correlation, 0.776, and SASI at the 1.5 order had the highest correlation, 0.778. In the three-band combinations, TBI1 at the 1.5 order had the highest R value of 0.785, TBI2 at the 1.25 order had the highest R value of 0.788, TBI3 at the 1.0 order had the highest R value of 0.813, TBI4 at the 1.0 order had the highest R value of 0.823, TBI5 at the 0.75 order had the highest R value of 0.806, TBI6 at the 1.0 order had the highest R value of 0.824, and TBI7 at the 1.5 order had the highest R value of 0.778. **Figures 9**, **10** show the correlation analysis of the optimal order for the two-dimensional and three-dimensional spectral indices. Compared to the spectral data from 0 to 2 orders, the two-dimensional and three-dimensional spectral indices substantially enhance the correlation between the original spectra and nitrogen content. Among them, the three-dimensional spectral indices generally outperform the two-dimensional indices, with TBI7 showing the highest correlation. This suggests that three-dimensional spectral indices are more effective in predicting nitrogen content.

**Figure 11:**
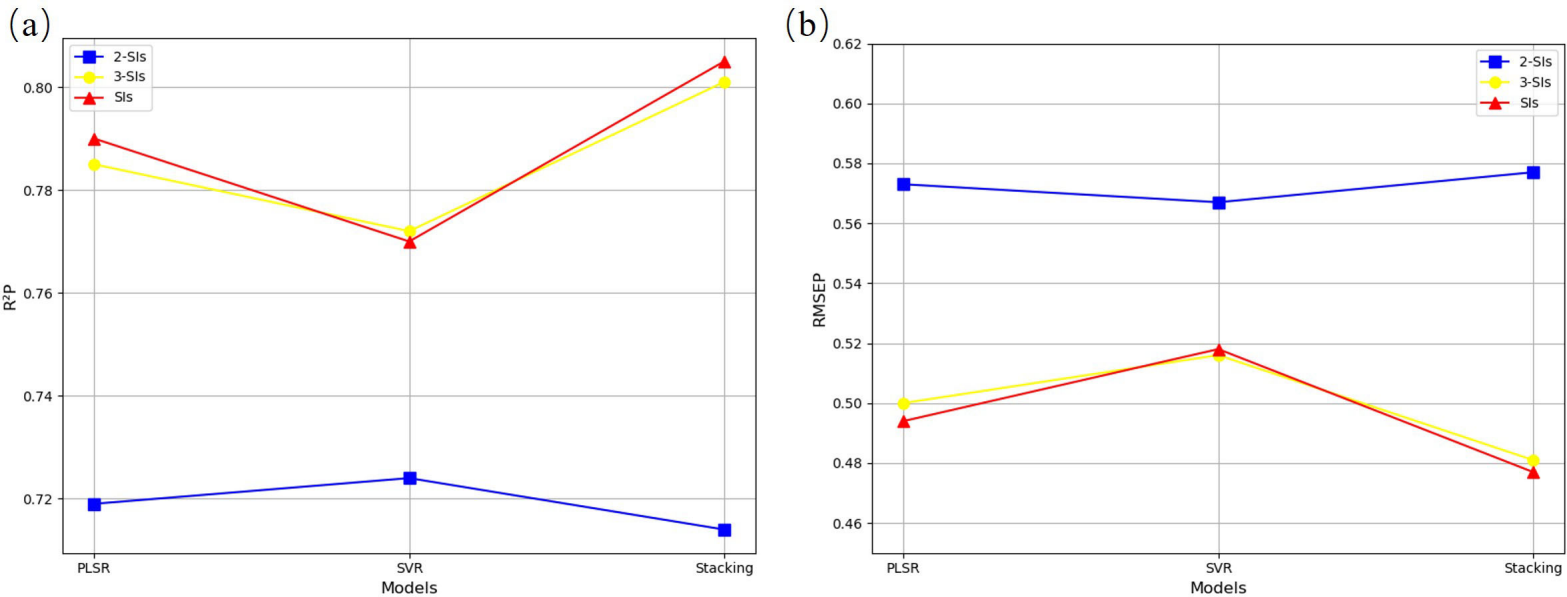
Accuracy statistics for N estimation with different spectral index combinations **(a)** R2P **(b)** RMSEP.

By combining two-dimensional spectral indices, three-dimensional spectral indices, and mixed two-dimensional-three-dimensional spectral indices as feature sets, the effects of different spectral index combinations on nitrogen content prediction were analyzed using PLSR, SVR, and Stacking models (**Figure 11**, **Supplementary Table 6**). The results show that the three-dimensional spectral indices significantly outperformed the two-dimensional spectral indices on the test set, especially in the Stacking model, where R²p increased from 0.719 to 0.785, and RMSEP decreased from 0.573 to 0.500. This further confirms that three-dimensional spectral indices are better at capturing the complex relationship between nitrogen content and spectral features. In the mixed spectral indices, the Stacking model exhibited the best performance with an R²p of 0.805 and an RMSEP of 0.477, clearly outperforming the individual applications of two-dimensional and three-dimensional spectral indices. The combination of two-dimensional and three-dimensional mixed spectral indices significantly improved the model’s prediction accuracy and generalization ability, demonstrating the advantages of feature combination.

### 3.4 Model training and performance analysis

**Figure 12:**
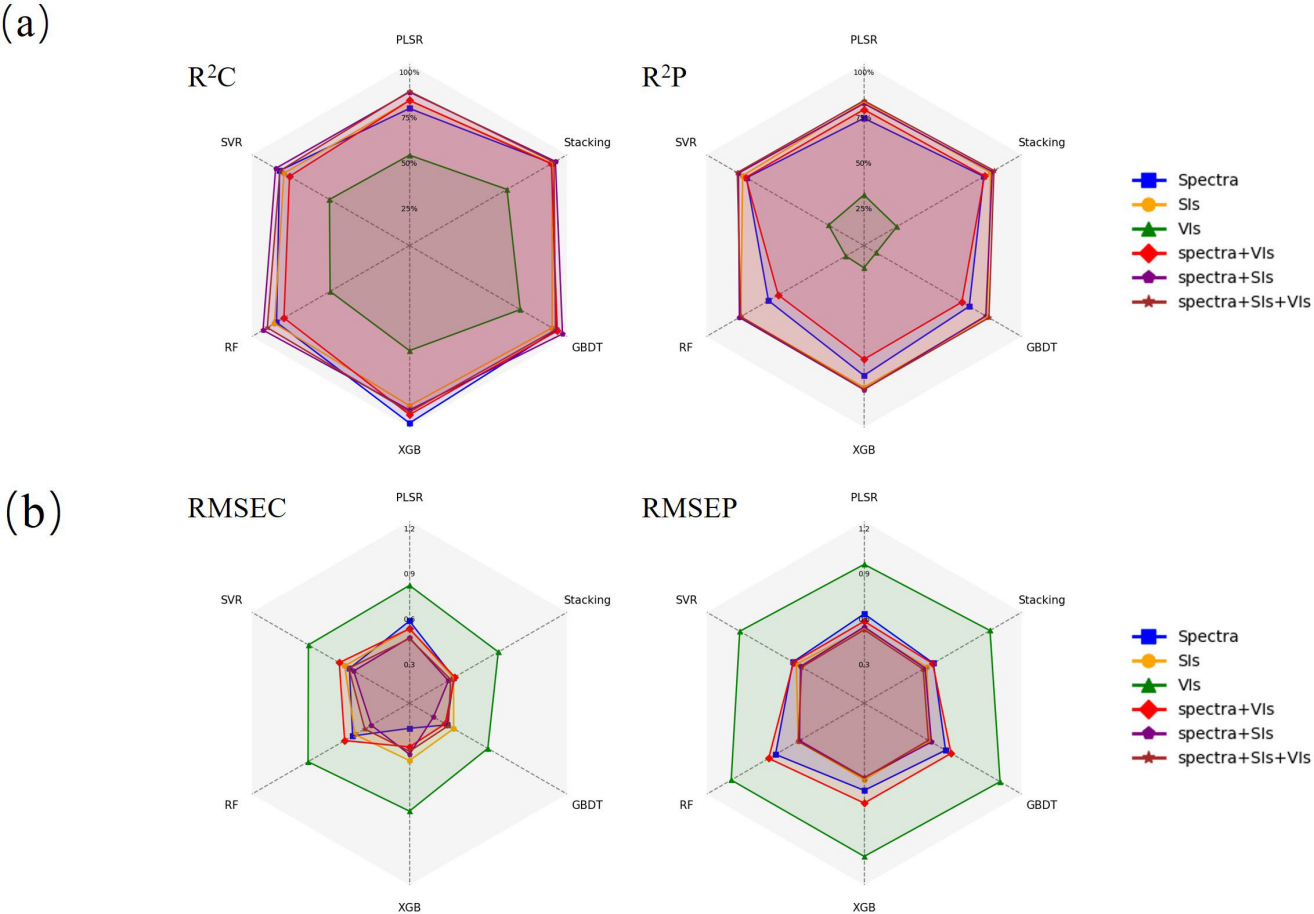
Performance of different machine learning models **(a)** R2 **(b)** RMSE.

In this study, we compared the nitrogen content prediction performance of different feature sets (Spectra, SIs, VIs, Spectra-VIs, Spectra-SIs, Spectra-Vis-SIs) and various models (PLSR, SVR, RF, GBDT, XGB, Stacking) (**Figure 12**, **Supplementary Table 7**). Firstly, the results of the GA-SPA (Spectra) feature set showed that XGB performed exceptionally well on the training set, with an R²c value of 0.977 and an RMSEC of only 0.166, demonstrating its strong fitting ability. However, XGB’s performance on the test set was less impressive, with an R²p of 0.715 and an RMSEP of 0.576, indicating significant overfitting and highlighting the model’s limited generalization capability as the main performance constraint.

In order to mitigate the overfitting issue observed with XGBoost, the Stacking model was employed. Stacking integrates predictions from multiple base learners, which helps to reduce the overfitting risk of any individual model, particularly XGBoost, by leveraging the diversity of base learners such as GBDT and SVR. This fusion of different models allows for more balanced predictions, improving the model’s ability to generalize to unseen data. The result was an improvement in generalization, as evidenced by the better performance of the Stacking model on the test set (R²p of 0.761 and RMSEP of 0.528).

Additionally, regularization techniques were applied to the XGBoost model to further control overfitting. Specifically, L2 regularization was used to penalize large coefficients, and techniques such as subsampling and column sampling were implemented to reduce model complexity and prevent overfitting to the training data. These regularization techniques, in combination with the Stacking approach, allowed the model to maintain high performance while avoiding overfitting, ensuring better generalization across all feature sets.

When the spectral index (SIs) feature set was introduced, the Stacking model still held the advantage, with an R²p of 0.805 and an RMSEP of 0.477, indicating that it outperformed other individual models, especially in terms of information integration. Although GBDT and XGB showed good fitting ability on the training set, their performance on the test set was not as stable as that of the Stacking model, which may be related to overfitting. Additionally, the VIs feature set performed relatively poorly across all models. Neither XGB nor Stacking could achieve R²p and RMSEP values comparable to those of other feature sets, suggesting that the VIs feature set lacks sufficient information content for nitrogen content prediction, which limits the model’s predictive performance. This further proves the limitations of using a single feature set for nitrogen content prediction, while fusion of feature sets can significantly improve model performance.

**Figure 13:**
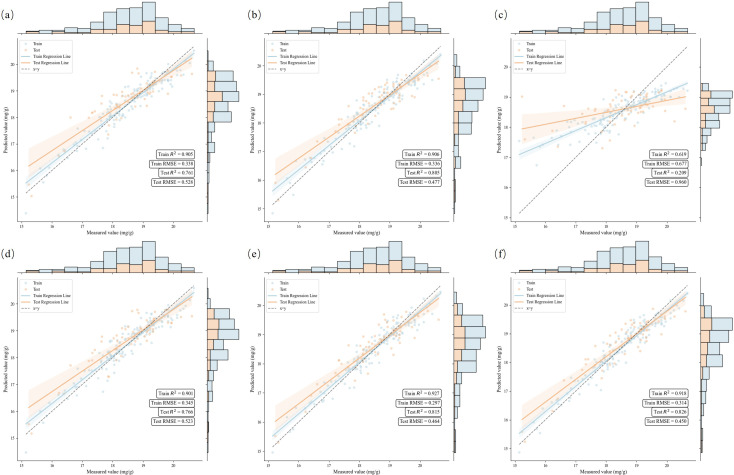
Scatter plot of the true and predicted nitrogen content values of stacking under different datasets.

**Figure 14:**
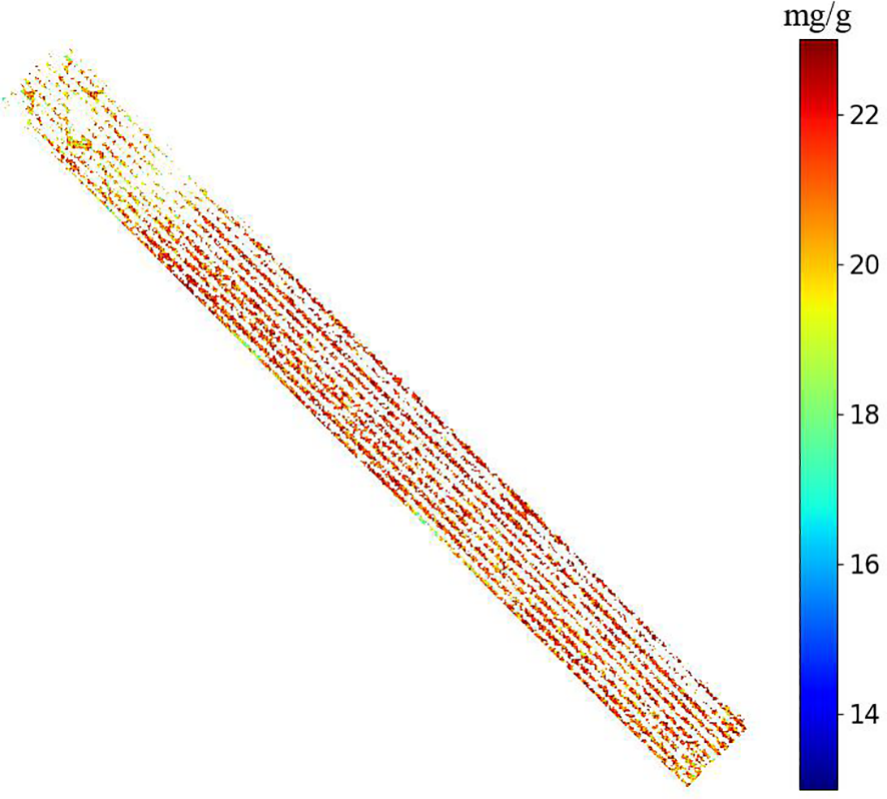
In the study area, a nitrogen content distribution map was generated using high-resolution unmanned aerial vehicle (UAV) hyperspectral images through the Spectra-Vis-Sis-Stacking method.

For the fused feature sets Spectra-VIs and Spectra-SIs, the performance of the Stacking model improved further. Particularly for the Spectra-SIs feature set, Stacking achieved an R²p of 0.815 and an RMSEP of 0.464, significantly outperforming other individual models. At this point, the combination of spectral bands and spectral indices provided more useful information, and the Stacking model, by integrating different base learners, was able to more effectively leverage this information, resulting in better performance on the test set. For the Spectra-Vis-SIs fused feature set, the Stacking model reached an R²p of 0.826 and an RMSEP of 0.450, which was the best performance among all feature sets, fully demonstrating the effectiveness of multi-source feature fusion. XGB and GBDT also performed well with this feature set, but the RMSEP of the Stacking model was slightly lower, showing its advantage in generalization ability. **Figure 13** displays scatter plots of the actual versus predicted nitrogen content values for the Stacking model across different datasets, further illustrating its outstanding performance in multi-source feature fusion. Meanwhile, **Figure 14** shows the successful generation of a nitrogen content distribution map for the study area using high-resolution UAV hyperspectral images and the Spectra-Vis-SIs-Stacking method, validating the effectiveness of this approach in practical applications.

A comparison of the results from different feature sets and models reveals that the Stacking model consistently outperforms other individual models across all feature sets, particularly for fused feature sets with strong information complementarity. In these cases, the Stacking model is better able to integrate the strengths of different base learners, thereby improving the accuracy of nitrogen content prediction. Fused feature sets (such as Spectra + VIs + SIs) combine different types of information, providing a more comprehensive feature representation, allowing the model to more accurately capture variations in nitrogen content. These results demonstrate that the complementarity of information is crucial for enhancing model performance. The original spectral bands provide detailed features, vegetation indices support biochemical information, and spectral indices reveal unique information that is highly correlated with nitrogen content. The fusion of these three sources not only strengthens the complementarity of information but also significantly improves the model’s predictive capability.

### 3.5 Model interpretability analysis

**Figure 15:**
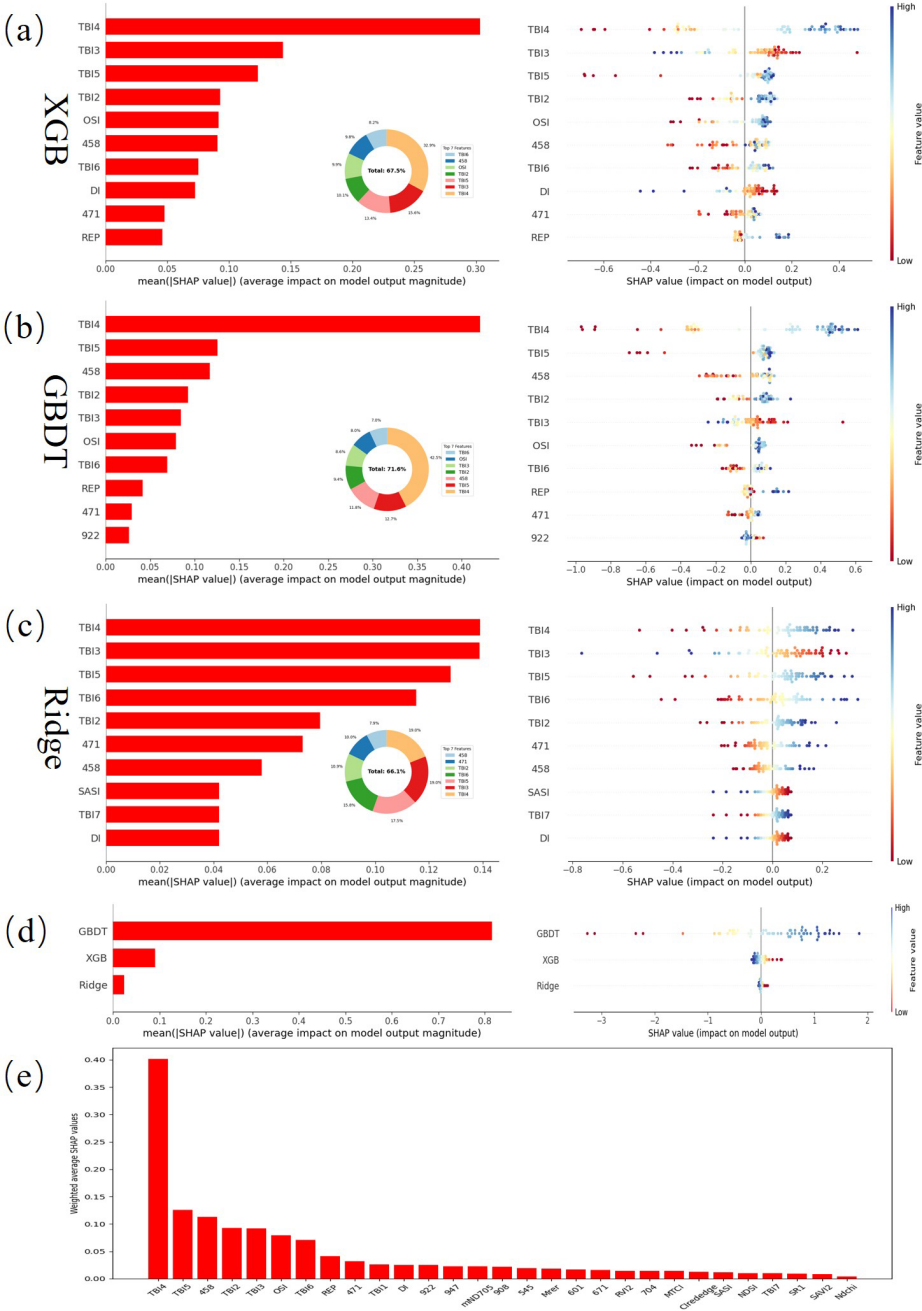
Shapely additive explanation for the optimal stacking model **(a)** Basic model-XGB **(b)** Basic model-GBDT **(c)** Basic model-Ridge **(d)** Meta model-Bayesian Ridge **(e)** The ranked graph of the weighted fused features.

In this section, we conducted a global analysis of feature importance based on SHAP values, aiming to reveal the role and contribution of each feature in predicting nitrogen content in maize. **Figure 15** displays the SHAP analysis results, showing that different spectral indices, vegetation indices, and specific spectral bands make significant contributions to nitrogen content prediction, with varying performance across base models (XGB, GBDT, Ridge) and the stacked meta-model (Bayesian Ridge).

First, the 3D spectral indices TBI4, TBI3, and TBI5 occupy significant positions in all base models. Particularly TBI4, which stands out in the XGB and GBDT models, contributing 32.9% and 42.5% respectively to the top seven most important features. This spectral band likely contains critical information reflecting the physiological status of the maize canopy, further validating the key role of the red-edge region in nitrogen content prediction. TBI6 and TBI2 also show considerable importance across the models, indicating their significant contribution to capturing nitrogen content features in the maize canopy. In contrast, the 2D spectral indices (DI, OSI, SASI) have lower contributions in the models, suggesting that their predictive ability is slightly inferior compared to the 3D spectral indices.

Among the base models, GBDT contributes the most in the SHAP analysis, particularly demonstrating strong nonlinear modeling capability in the contribution of TBI4. Although XGB and Ridge models also contribute to other bands and spectral indices, their performance in capturing complex patterns is relatively weaker. The meta-model (Bayesian Ridge) further enhances overall predictive ability by optimizing the outputs of the base models, with GBDT’s contribution still dominating, reinforcing its central role in nitrogen content prediction.

Certain specific bands, such as 471 nm and 458 nm, also exhibit high contributions in the XGB and GBDT models. These bands, located in the visible light region, are particularly sensitive to chlorophyll absorption, making them crucial for capturing nitrogen-related physiological features. However, when used alone, their predictive capability is somewhat limited and typically requires combination with spectral indices or vegetation indices to achieve more accurate predictions.

Compared to spectral indices and bands, the contribution of vegetation indices is relatively weaker. In this study, REP only ranked within the top ten in XGB and GBDT, suggesting that while vegetation indices can provide supplementary information in some cases, they do not perform as well as spectral indices and bands in nitrogen content prediction. Particularly in nonlinear modeling processes, the contribution of vegetation indices is more limited and typically does not directly reflect complex nitrogen content features.

Overall, SHAP analysis reveals the dominant role of spectral indices, particularly 3D spectral indices, in maize nitrogen content prediction. Among the top ten features, spectral indices occupy seven positions, further validating their key role in the prediction process. Although feature bands and vegetation indices play complementary roles under certain conditions, they primarily assist in detail capture and nonlinear modeling. Ultimately, the outstanding performance of TBI4 and its associated bands in all features confirms the core position of the red-edge region in nitrogen content prediction.

Through the weighted fusion of base models in the Stacking approach, the model maximizes the complementarity of multi-level features, further enhancing overall predictive accuracy. Despite variations in feature selection performance across different base models, spectral indices, particularly 3D spectral indices, remain the most critical features for predicting maize nitrogen content.

## 4 Discussion

### 4.1 Effectiveness of feature selection strategies

The results of this study are consistent with previous research highlighting the effectiveness of hybrid feature selection strategies in hyperspectral analysis. For example Qin et al., 2025, and Liu et al., 2024 demonstrated that combinations such as VISSA-CARS and CARS-SPA can significantly improve prediction accuracy by reducing redundant spectral information. However, most existing studies primarily focus on linear or semi-linear selection strategies, which may not fully capture the complex nonlinear relationships in hyperspectral data.

In contrast, the GA-SPA framework proposed in this study integrates global optimization and local refinement, enabling a more effective exploration of the high-dimensional spectral space. This dual-stage mechanism not only improves feature relevance but also enhances the stability of the selected feature subset. More importantly, compared with traditional hybrid methods, the GA-SPA approach demonstrates stronger adaptability when integrated into nonlinear ensemble models such as stacking, highlighting its advantage in complex modeling scenarios.

Therefore, this study extends existing research by demonstrating that hybrid feature selection strategies can be further optimized through the integration of global–local search mechanisms, providing a more robust solution for hyperspectral feature reduction in nitrogen estimation tasks.

### 4.2 Insights on fractional order derivatives and spectral indices

The results of this study demonstrate that the combination of FOD and Spectral Indices (SIs) significantly improves the prediction accuracy of maize nitrogen content. Specifically, within the range of fractional orders from 0.75 to 1.5, the correlation between spectral data and nitrogen content is notably enhanced, reaching its optimal performance at an order of 1.5. This phenomenon suggests that FOD is capable of capturing finer spectral variations while strengthening the synergistic effects between different spectral dimensions, thereby improving the model’s sensitivity to changes in nitrogen content (Xiao et al., 2024). Compared with traditional integer-order derivatives commonly used in hyperspectral analysis, fractional-order differentiation provides greater flexibility in capturing subtle spectral variations and enhancing signal-to-noise characteristics.

Three-dimensional spectral indices also show distinct advantages in this study. Compared to traditional two-dimensional indices, 3D indices, by incorporating an additional spectral dimension, are able to capture more information related to the plant’s nitrogen physiological status (Yang et al., 2022). Although two-dimensional indices are somewhat effective in reflecting chlorophyll content, they are limited in fully characterizing the complex distribution and metabolic processes of nitrogen within the plant.

Previous studies have mainly focused on two-band or vegetation index-based approaches, which often provide limited representation of complex biochemical interactions within the canopy (Sun et al., 2024). In contrast, the introduction of three-dimensional spectral indices in this study enables the integration of multiple spectral interactions, thereby improving the characterization of nitrogen-related physiological processes.

In conclusion, the combination of FOD and spectral indices not only optimizes the model’s predictive capability but also provides a more comprehensive and detailed perspective for nitrogen detection (Yang et al., 2022; Li et al., 2023a; Tang et al., 2025). This study further extends existing research by systematically integrating fractional-order transformation with multi-dimensional spectral index construction, which has rarely been explored in maize nitrogen estimation.

### 4.3 Synergistic effect of multi-source feature fusion and ensemble learning models

This study significantly improves maize nitrogen content prediction accuracy through multi-source feature fusion and stacked ensemble learning models. Firstly, a systematic screening of vegetation indices revealed that traditional vegetation indices performed inadequately in predicting maize nitrogen content. Despite improvements using various statistical and machine learning methods, the optimal vegetation index still showed insufficient predictive accuracy (R² = 0.27). This highlights the limitations of vegetation indices in predicting specific crop and physiological indicators such as nitrogen content. The main issue stems from the general nature of vegetation index design, which exhibits low sensitivity to nitrogen changes, particularly when nitrogen stress has not yet significantly affected pigment levels, weakening its indicative role. Therefore, relying solely on vegetation indices is insufficient to accurately capture changes in nitrogen content.

This finding is consistent with previous studies, which have reported that vegetation indices alone often show limited sensitivity to nitrogen variations, especially under moderate nitrogen stress conditions (Li et al., 2023b). However, the screening of vegetation indices provided important insights for feature fusion. The selected “optimal” vegetation index, although with weaker sensitivity, acted as a robust indicator within an existing knowledge framework and played a crucial role in subsequent fusion, enhancing the effectiveness of other features. When combined with GA-SPA optimized bands and FOD spectral indices, this complementarity enhanced the model’s ability to locate and be sensitive to nitrogen content.

The advantage of feature fusion lies in integrating complementary information from different sources. The GA-SPA bands provide precise nitrogen-related signals, while the FOD spectral indices enhance the model’s sensitivity by capturing subtle spectral changes. The optimized vegetation indices contribute a robust physiological background for the model. By merging these features, the model operates in a more comprehensive feature space, avoiding errors that may arise from reliance on a single feature. Compared with single-feature modeling approaches widely used in previous studies, feature fusion strategies allow the integration of complementary information from spectral bands, vegetation indices, and derived spectral indices, resulting in more robust and accurate predictions.

The stacked ensemble learning model further enhances the effectiveness of feature fusion. By dynamically adjusting feature weights, the meta-learner optimizes the integration process, ensuring the relevance of the most significant features. When the contribution of vegetation indices is low, the model adaptively reduces their weight and relies more on the more representative FOD and GA-SPA bands, improving prediction stability and accuracy. This process of model fusion also helps mitigate overfitting risks from individual base learners. Specifically, while XGBoost demonstrated exceptional training performance, the stacking model effectively balanced its overfitting tendencies by combining predictions from other models like GBDT and SVR, which had more stable performance across the test set. Furthermore, regularization techniques, such as L2 regularization and subsampling, were applied to XGBoost to further control overfitting and improve generalization. These techniques helped to prevent the model from becoming overly complex and ensured more reliable predictions, especially when integrated into the stacked ensemble framework.

In conclusion, multi-source feature fusion and stacked ensemble learning models effectively overcome the limitations of individual features. Through the complementarity and dynamic integration of information, they significantly enhance the prediction accuracy of maize nitrogen content, offering new approaches for remote sensing monitoring of industrial crops and agricultural data analysis in the future.

### 4.4 Limitations and future outlook

This study effectively improves the accuracy of maize nitrogen content detection based on hyperspectral data through multi-source feature fusion and a stacked ensemble learning framework. Our core contribution lies in the introduction and validation of the GA-SPA hybrid feature selection method, which not only reduces redundant features but also enhances the predictive capability of the model when processing hyperspectral data. By combining FOD and three-dimensional spectral indices, the study confirms that three-dimensional indices are superior to traditional two-dimensional spectral indices in extracting potential spectral features, offering a new technological pathway for crop nitrogen monitoring. Furthermore, by integrating spectral information from different sources, this study optimized the prediction model and used the SHAP explainability tool to provide transparent physical interpretations of the model’s decisions, thus enhancing the model’s credibility and practical value.

Despite these positive results, several specific limitations must be acknowledged. First, the model was developed and validated using only one maize variety. Spectral responses to nitrogen stress may vary significantly across varieties due to differences in leaf structure, pigment composition, and canopy architecture, which could substantially limit the model’s generalization ability. To address this issue in future studies, we plan to include at least three representative maize varieties with distinct leaf angles and chlorophyll backgrounds in the experimental design. A leave-one-variety-out cross-validation strategy will be employed to quantitatively assess cross-variety generalization, and domain adaptation techniques will be explored using a small amount of target-variety data for variety-specific calibration.

Second, the current experiment involved only four nitrogen application rates and lacked sufficient spatial replication in the field. This may have allowed the model to learn spurious correlations related to micro-environmental heterogeneity rather than genuine nitrogen-induced spectral signals, thereby compromising robustness in new environments. Consequently, future field experiments will include at least six nitrogen gradients ranging from severe deficiency to excess, with a randomized complete block design comprising five or more replicates per treatment. Additionally, soil background spectra will be collected at each sampling point to enable background normalization, and difference-based spectral indices will be used to mitigate environmental interference.

Third, data were collected only at the maize tasseling stage, which provides a static snapshot and fails to capture the dynamic processes of nitrogen uptake, remobilization, and dilution over the growing season. This restricts the model’s applicability for early warning or in-season management. In future work, we will conduct time-series sampling across at least four key growth stages—jointing, bell-bottom, tasseling, and filling—to capture temporal nitrogen dynamics. These data will be used to train growth-stage-specific models and to develop a temporal transfer learning framework. Specifically, recurrent neural networks (RNN) or long short-term memory networks (LSTM) will be implemented to model nitrogen content trajectories, enabling dynamic prediction and early detection of nitrogen stress.

In summary, to collectively address the above limitations, our prioritized research roadmap includes designing a multi-variety, multi-nitrogen-gradient, multi-replicate field experiment with time-series sampling, establishing a benchmark dataset for cross-environment model transfer, and integrating growth-stage-aware ensemble models with recurrent architectures. These steps will systematically enhance model generalizability, robustness, and practical utility for precision nitrogen management.

## 5 Conclusion

This study developed and validated a framework based on UAV hyperspectral technology, integrating multi-source feature optimization and stacked ensemble learning, for accurate and interpretable detection of maize nitrogen content. The main findings of the study are as follows:

1. The hybrid feature selection strategy (GA-SPA) outperforms individual methods in selecting informative bands from raw hyperspectral data, effectively addressing redundancy and collinearity issues in high-dimensional data.
2. The introduction of FOD, particularly in constructing three-dimensional spectral indices, has been demonstrated as a key innovation. It systematically outperforms traditional vegetation indices and two-dimensional indices in capturing subtle spectral responses related to nitrogen stress.
3. The fusion of multi-source features (optimized bands, vegetation indices, and spectral indices) combined with the stacked ensemble model (XGBoost, GBDT, and Ridge as base models, Bayesian Ridge as the meta-model) achieved the highest prediction accuracy, highlighting the core value of complementary fusion across different information sources.
4. SHAP explainability analysis not only enhanced the model’s transparency by identifying the most significant features contributing to the prediction, but also successfully linked data-driven machine learning decisions with crop physiological mechanisms, thereby improving the credibility of the results.

The methodology proposed in this study provides a reliable technical pathway for nitrogen nutrition monitoring in industrial maize. The developed feature optimization strategies and interpretable ensemble models are expected to be integrated into intelligent agricultural decision-making systems, offering effective solutions for on-demand nitrogen fertilizer management and promoting the sustainable development of bio-based crop production.

## Data Availability

The raw data supporting the conclusions of this article will be made available by the authors, without undue reservation.

## References

[B1] Ahmed T. Wijewardane N. K. Lu Y. Jones D. S. Kudenov M. Williams C. . (2024). Advancing sweetpotato quality assessment with hyperspectral imaging and explainable artificial intelligence. Comput. Electron. Agric. 220, 108855. doi: 10.1016/j.compag.2024.108855. PMID: 38826717

[B2] Barbosa B. D. S. Costa L. Ampatzidis Y. Vijayakumar V. dos Santos L. M. (2021). UAV-based coffee yield prediction utilizing feature selection and deep learning. Smart. Agric. Technol. 1, 100010. doi: 10.1016/j.atech.2021.100010. PMID: 38826717

[B3] Blackburn G. A. (1998). Quantifying chlorophylls and caroteniods at leaf and canopy scales: An evaluation of some hyperspectral approaches. Remote Sens. Environ. 66, 273–285. doi: 10.1016/S0034-4257(98)00059-5

[B4] Cao C. Wang T. Gao M. Li Y. Li D. Zhang H. (2021). Hyperspectral inversion of nitrogen content in maize leaves based on different dimensionality reduction algorithms. Comput. Electron. Agric. 190, 106461. doi: 10.1016/j.compag.2021.106461. PMID: 38826717

[B5] Castro K. L. Sanchez-Azofeifa G. A. (2008). Changes in spectral properties, chlorophyll content and internal mesophyll structure of senescing Populus balsamifera and Populus tremuloides leaves. Sensors 8, 51–69. doi: 10.3390/s8010051. PMID: 27879696 PMC3681139

[B6] Chen J. M. (1996). Evaluation of vegetation indices and a modified simple ratio for boreal applications. Can. J. Remote Sens. 22, 229–242. doi: 10.1080/07038992.1996.10855178. PMID: 37339054

[B7] Chen X. Lv X. Ma L. Chen A. Zhang Q. Zhang Z. (2022). Optimization and validation of hyperspectral estimation capability of cotton leaf nitrogen based on SPA and RF. Remote Sens. 14, 5201. doi: 10.3390/rs14205201. PMID: 30654563

[B8] Cheng J.-H. Sun D.-W. (2017). Partial least squares regression (PLSR) applied to NIR and HSI spectral data modeling to predict chemical properties of fish muscle. Food Eng. Rev. 9, 36–49. doi: 10.1007/s12393-016-9147-1. PMID: 30311153

[B9] Concepcion J. S. Noble A. D. Thompson A. M. Dong Y. Olson E. L. (2025). Genomic and hyperspectral imaging-based prediction blending enables selection for reduced deoxynivalenol content in wheat grains. G3.: Genes. Genomes. Genet. 15, jkaf176. doi: 10.1093/g3journal/jkaf176. PMID: 40795041 PMC12506668

[B10] Dash J. Curran P. J. (2007). Evaluation of the MERIS terrestrial chlorophyll index (MTCI). Adv. Space. Res. 39, 100–104. doi: 10.1016/j.asr.2006.02.034. PMID: 38826717

[B11] Daughtry C. S. Walthall C. Kim M. De Colstoun E. B. McMurtrey Iii J. (2000). Estimating corn leaf chlorophyll concentration from leaf and canopy reflectance. Remote Sens. Environ. 74, 229–239. doi: 10.1016/S0034-4257(00)00113-9

[B12] Feng W. Guo B.-B. Zhang H.-Y. He L. Zhang Y.-S. Wang Y.-H. . (2015). Remote estimation of above ground nitrogen uptake during vegetative growth in winter wheat using hyperspectral red-edge ratio data. Field Crops Res. 180, 197–206. doi: 10.1016/j.fcr.2015.05.020. PMID: 38826717

[B13] Fitzgerald G. Rodriguez D. Christensen L. Belford R. Sadras V. Clarke T. (2006). Spectral and thermal sensing for nitrogen and water status in rainfed and irrigated wheat environments. Precis. Agric. 7, 233–248. doi: 10.1007/s11119-006-9011-z. PMID: 30311153

[B14] Floor F. Jordan C. Jordan C. Rico P. Center N. Piedras R. (1969). Derivation of leaf-area index from quality of light on the derivation of leaf-area index from quality of light on the forest floor. Ecology 50 (4). doi: 10.2307/1936256

[B15] Gitelson A. A. Kaufman Y. J. Merzlyak M. N. (1996). Use of a green channel in remote sensing of global vegetation from EOS-MODIS. Remote Sens. Environ. 58, 289–298. doi: 10.1016/S0034-4257(96)00072-7

[B16] Gitelson A. A. Keydan G. P. Merzlyak M. N. (2006). Three‐band model for noninvasive estimation of chlorophyll, carotenoids, and anthocyanin contents in higher plant leaves. Geophys. Res. Lett. 33 (11). doi: 10.1029/2006GL026457. PMID: 29143083

[B17] Gitelson A. Merzlyak M. N. (1994). Quantitative estimation of chlorophyll-a using reflectance spectra: Experiments with autumn chestnut and maple leaves. J. Photochem. Photobiol. B. Biol. 22, 247–252. doi: 10.1016/1011-1344(93)06963-4

[B18] Guyot G. Baret F. Major D. J. (1988). High spectral resolution: determination of spectral shifts between the red and near infrared. Int. Arch. Photogrammetry. Remote Sens. 27, 11. Available online at: https://hal.inrae.fr/hal-02717525 42066341

[B19] Huang H.-Y. Ding Q.-D. Zhang J.-H. Pan X. Zhou Y.-H. Jia K.-L. (2024). Ground-based hyperspectral inversion of salinization and alkalinization of different soil layers in farmland in Yinbei area, Ningxia, China. J. Appl. Ecol. 35, 3073–3084. 39925064 10.13287/j.1001-9332.202411.017

[B20] Huang L. Liu Y. Huang W. Dong Y. Ma H. Wu K. . (2022). Combining random forest and XGBoost methods in detecting early and mid-term winter wheat stripe rust using canopy level hyperspectral measurements. Agriculture 12, 74. doi: 10.3390/agriculture12010074. PMID: 30654563

[B21] Huete A. R. (1988). A soil-adjusted vegetation index (SAVI). Remote Sens. Environ. 25, 295–309. doi: 10.1016/0034-4257(88)90106-X

[B22] Ji S. Gu C. Xi X. Zhang Z. Hong Q. Huo Z. . (2022). Quantitative monitoring of leaf area index in rice based on hyperspectral feature bands and ridge regression algorithm. Remote Sens. Environ. 14, 2777. doi: 10.3390/rs14122777. PMID: 30654563

[B23] Jiang Z. Huete A. R. Didan K. Miura T. (2008). Development of a two-band enhanced vegetation index without a blue band. Remote Sens. Environ. 112, 3833–3845. doi: 10.1016/j.rse.2008.06.006. PMID: 38826717

[B24] Jin J. Arief Pratama B. Wang Q. (2020). Tracing leaf photosynthetic parameters using hyperspectral indices in an alpine deciduous forest. Remote Sens. 12, 1124. doi: 10.3390/rs12071124

[B25] Jin X. Song K. Du J. Liu H. Wen Z. (2017). Comparison of different satellite bands and vegetation indices for estimation of soil organic matter based on simulated spectral configuration. Agric. For. Meteorol. 244, 57–71. doi: 10.1016/j.agrformet.2017.05.018. PMID: 38826717

[B26] Karyoti A. Giannoulis K. D. Bartzialis D. Hatzigiannakis E. Skoufogianni E. Danalatos N. G. (2021). Green manuring for low-input irrigated maize cultivation as an energy crop in Mediterranean climates. Int. J. Plant Prod. 15, 563–575. doi: 10.1007/s42106-021-00165-1. PMID: 30311153

[B27] Kuglarz K. Bury M. Kasprzycka A. Lalak-Kańczugowska J. (2020). Effect of nitrogen fertilization on the production of biogas from sweet sorghum and maize biomass. Environ. Technol. doi: 10.1080/09593330.2019.1584251. PMID: 30767620

[B28] Lao C. Chen J. Zhang Z. Chen Y. Ma Y. Chen H. . (2021). Predicting the contents of soil salt and major water-soluble ions with fractional-order derivative spectral indices and variable selection. Comput. Electron. Agric. 182, 106031. doi: 10.1016/j.compag.2021.106031. PMID: 38826717

[B29] Lemaire G. Jeuffroy M.-H. Gastal F. (2008). Diagnosis tool for plant and crop N status in vegetative stage: Theory and practices for crop N management. Eur. J. Agron. 28, 614–624. doi: 10.1016/j.eja.2008.01.005. PMID: 38826717

[B30] Li J. Cheng K. Wang S. Morstatter F. Trevino R. P. Tang J. . (2017). Feature selection: A data perspective. ACM Comput. Surv. 50, 1–45. doi: 10.1145/3136625

[B31] Li C. Li X. Meng X. Xiao Z. Wu X. Wang X. . (2023a). Hyperspectral estimation of nitrogen content in wheat based on fractional difference and continuous wavelet transform. Agriculture 13, 1017. doi: 10.3390/agriculture13051017. PMID: 30654563

[B32] Li F. Mistele B. Hu Y. Chen X. Schmidhalter U. (2013). Comparing hyperspectral index optimization algorithms to estimate aerial N uptake using multi-temporal winter wheat datasets from contrasting climatic and geographic zones in China and Germany. Agric. For. Meteorol. 180, 44–57. doi: 10.1016/j.agrformet.2013.05.003. PMID: 38826717

[B33] Li S. Sun L. Tian Y. Lu X. Fu Z. Lv G. . (2024). Research on non-destructive identification technology of rice varieties based on HSI and GBDT. Infrared. Phys. Technol. 142, 105511. doi: 10.1016/j.infrared.2024.105511. PMID: 38826717

[B34] Li Z. Zhou X. Cheng Q. Fei S. Chen Z. (2023b). A machine-learning model based on the fusion of spectral and textural features from UAV multi-sensors to analyse the total nitrogen content in winter wheat. Remote Sens. 15, 2152. doi: 10.3390/rs15082152. PMID: 30654563

[B35] Lin Y. Gao J. Tu Y. Zhang Y. Gao J. (2024). Estimating low concentration heavy metals in water through hyperspectral analysis and genetic algorithm-partial least squares regression. Sci. Tot. Environ. 916, 170225. doi: 10.1016/j.scitotenv.2024.170225. PMID: 38246365

[B36] Lin J. Meng Q. Wu Z. Pei S. Tian P. Huang X. . (2023). Nondestructive detection of mango soluble solid content in hyperspectral imaging based on multi-combinatorial feature wavelength selection. Acta Alimentaria. 52, 401–412. doi: 10.1556/066.2023.00014

[B37] Liu X. Zhang S. Chen S. Tuo Y. Peng K. Tan S. . (2024). Predicting leaf nitrogen content of coffee trees using the canopy hyperspectral reflectance feature bands, vegetation index and machine learning. Int. J. Remote Sens. 45, 8471–8498. doi: 10.1080/01431161.2024.2402005. PMID: 37339054

[B38] Ma L. Chen X. Zhang Q. Lin J. Yin C. Ma Y. . (2022). Estimation of nitrogen content based on the hyperspectral vegetation indexes of interannual and multi-temporal in cotton. Agronomy 12, 1319. doi: 10.3390/agronomy12061319. PMID: 30654563

[B39] Okab S. I. Abed Z. A. (2022). Effect of nitrogen fertilizers on growth and yield traits of maize: effect of nitrogen fertilizers on growth and yield traits of maize. Iraqi. J. Market. Res. Consumer. Prot. 14, 40–49. Available online at: https://jmracpc.uobaghdad.edu.iq/index.php/IJMRCP/article/view/301

[B40] Qi J. Chehbouni A. Huete A. R. Kerr Y. H. Sorooshian S. (1994). A modified soil adjusted vegetation index. Remote Sens. Environ. 48, 119–126. doi: 10.1016/0034-4257(94)90134-1

[B41] Qiao M. Cui T. Xia G. Xu Y. Li Y. Fan C. . (2024). Integration of spectral and image features of hyperspectral imaging for quantitative determination of protein and starch contents in maize kernels. Comput. Electron. Agric. 218, 108718. doi: 10.1016/j.compag.2024.108718. PMID: 38826717

[B42] Qin A. Sun J. Zhu X. Li M. Li C. Wang L. . (2025). The yield estimation of apple trees based on the best combination of hyperspectral sensitive wavelengths algorithm. Sustainability 17 (2). doi: 10.3390/su17020518. PMID: 30654563

[B43] Rawal A. Hartemink A. Zhang Y. Wang Y. Lankau R. A. Ruark M. D. (2024). Visible and near-infrared spectroscopy predicted leaf nitrogen contents of potato varieties under different growth and management conditions. Precis. Agric. 25, 751–770. doi: 10.1007/s11119-023-10091-z. PMID: 30311153

[B44] Rondeaux G. Steven M. Baret F. (1996). Optimization of soil-adjusted vegetation indices. Remote Sens. Environ. 55, 95–107. doi: 10.1016/0034-4257(95)00186-7

[B45] Rouse J. (1973). Monitoring the vernal advancement and retrogradation of natural vegetation. NASA/GSFCT. Type. II. Rep.

[B46] Ruan G. Schmidhalter U. Yuan F. Cammarano D. Liu X. Tian Y. . (2023). Exploring the transferability of wheat nitrogen status estimation with multisource data and Evolutionary Algorithm-Deep Learning (EA-DL) framework. Eur. J. Agron. 143, 126727. doi: 10.1016/j.eja.2022.126727. PMID: 38826717

[B47] Sellami M. H. Albrizio R. Čolović M. Hamze M. Cantore V. Todorovic M. . (2022). Selection of hyperspectral vegetation indices for monitoring yield and physiological response in sweet maize under different water and nitrogen availability. Agronomy 12, 489. doi: 10.3390/agronomy12020489. PMID: 30654563

[B48] Sharifi A. Felegari S. (2023). Remotely sensed normalized difference red-edge index for rangeland biomass estimation. Aircraft. Eng. Aerospace. Technol. 95, 1128–1136. doi: 10.1108/AEAT-07-2022-0199. PMID: 35579975

[B49] Shi D. Shrestha R. K. Obaid H. Elsayed N. S. Zhong S. Hashimi M. H. . (2023). Valorization of nitrogen-rich melamine as a nitrogen source in the production of maize (Zea mays L.). Ind. Crops Prod. 199, 116770. doi: 10.1016/j.indcrop.2023.116770. PMID: 38826717

[B50] Sun Q. Chen L. Gu X. Zhang S. Dai M. Zhou J. . (2023). Estimation of canopy nitrogen nutrient status in lodging maize using unmanned aerial vehicles hyperspectral data. Ecol. Inf. 78, 102315. doi: 10.1016/j.ecoinf.2023.102315. PMID: 38826717

[B51] Sun T. Li Z. Wang Z. Liu Y. Zhu Z. Zhao Y. . (2024). Monitoring of nitrogen concentration in soybean leaves at multiple spatial vertical scales based on spectral parameters. Plants 13, 140. doi: 10.3390/plants13010140. PMID: 38202447 PMC10780363

[B52] Tang Z. Lu J. Sun T. Xiang Y. Zhang X. Li Z. . (2025). Nitrogen nutritional diagnosis of winter oilseed rape (Brassica napus L.) using fractional-order derivative hyperspectral indices: Field evaluation of dual nitrogen nutritional indices. Ind. Crops Prod. 235, 121660. doi: 10.1016/j.indcrop.2025.121660. PMID: 38826717

[B53] Wang J. Ding J. Yu D. Ma X. Zhang Z. Ge X. . (2019). Capability of Sentinel-2 MSI data for monitoring and mapping of soil salinity in dry and wet seasons in the Ebinur Lake region, Xinjiang, China. Geoderma 353, 172–187. doi: 10.1016/j.geoderma.2019.06.040. PMID: 38826717

[B54] Wang Y. Janz B. Engedal T. de Neergaard A. (2017). Effect of irrigation regimes and nitrogen rates on water use efficiency and nitrogen uptake in maize. Agric. Water Manage. 179, 271–276. doi: 10.1016/j.agwat.2016.06.007. PMID: 38826717

[B55] Wang R. Tuerxun N. Zheng J. (2024). Improved estimation of SPAD values in walnut leaves by combining spectral, texture, and structural information from UAV-based multispectral image. Sci. Hortic. 328, 112940. doi: 10.1016/j.scienta.2024.112940. PMID: 38826717

[B56] Xiao B. Li S. Dou S. He H. Fu B. Zhang T. . (2024). Comparison of leaf chlorophyll content retrieval performance of citrus using FOD and CWT methods with field-based full-spectrum hyperspectral reflectance data. Comput. Electron. Agri. 217, 108559. doi: 10.1016/j.compag.2023.108559. PMID: 41416194

[B57] Xuan F. Su W. Chen Z. Huang X. Zhai W. Li X. . (2025). Performance of stacking machine learning and volume model for improving corn above ground biomass prediction. Plant Phenomics. 7 (3), 100068. doi: 10.1016/j.plaphe.2025.100068. PMID: 41416194 PMC12709962

[B58] Xue L. Cao W. Luo W. Dai T. Zhu Y. (2004). Monitoring leaf nitrogen status in rice with canopy spectral reflectance. Agron. J. 96, 135–142. doi: 10.2134/agronj2004.1350

[B59] Yang C. Feng M. Song L. Jing B. Xie Y. Wang C. . (2022). Study on hyperspectral monitoring model of soil total nitrogen content based on fractional-order derivative. Comput. Electron. Agric. 201, 107307. doi: 10.1016/j.compag.2022.107307. PMID: 38826717

[B60] Yang D. Hu J. (2024). A detection method of oil content for maize kernels based on CARS feature selection and deep sparse autoencoder feature extraction. Ind. Crops Prod. 222, 119464. doi: 10.1016/j.indcrop.2024.119464. PMID: 38826717

[B61] Yang H. Yin H. Li F. Hu Y. Yu K. (2023). Machine learning models fed with optimized spectral indices to advance crop nitrogen monitoring. Field Crops Res. 293, 108844. doi: 10.1016/j.fcr.2023.108844. PMID: 38826717

[B62] Zarco-Tejada P. J. Miller J. R. Noland T. L. Mohammed G. H. Sampson P. H. (2002). Scaling-up and model inversion methods with narrowband optical indices for chlorophyll content estimation in closed forest canopies with hyperspectral data. IEEE Trans. Geosci. Remote Sens. 39, 1491–1507. doi: 10.1109/36.934080

[B63] Zhong K. Li Y. Huan W. Weng X. Wu B. Chen Z. . (2024). A novel near infrared spectroscopy analytical strategy for soil nutrients detection based on the DBO-SVR method. Spectrochimica. Acta Part. A. MolecularBiomolecular. Spectrosc. 315, 124259. doi: 10.1016/j.saa.2024.124259. PMID: 38636428

[B64] Ziadi N. Bélanger G. Gastal F. Claessens A. Lemaire G. Tremblay N. (2009). Leaf nitrogen concentration as an indicator of corn nitrogen status. Agron. J. 101, 947–957. doi: 10.2134/agronj2008.0172x

