# Supplementary material for: Hyperspectral data-driven corn nitrogen monitoring: application and interpretability analysis of multi-source feature optimization and stacked ensemble learning methods
Source: Front Plant Sci. 2026 May 21;17:1734394. doi: 10.3389/fpls.2026.1734394 (PMC13233678; doi:10.3389/fpls.2026.1734394)
Supplement: Supplementary file 1 [file DataSheet1.docx]

Supplementary Material

## Supplementary Figures


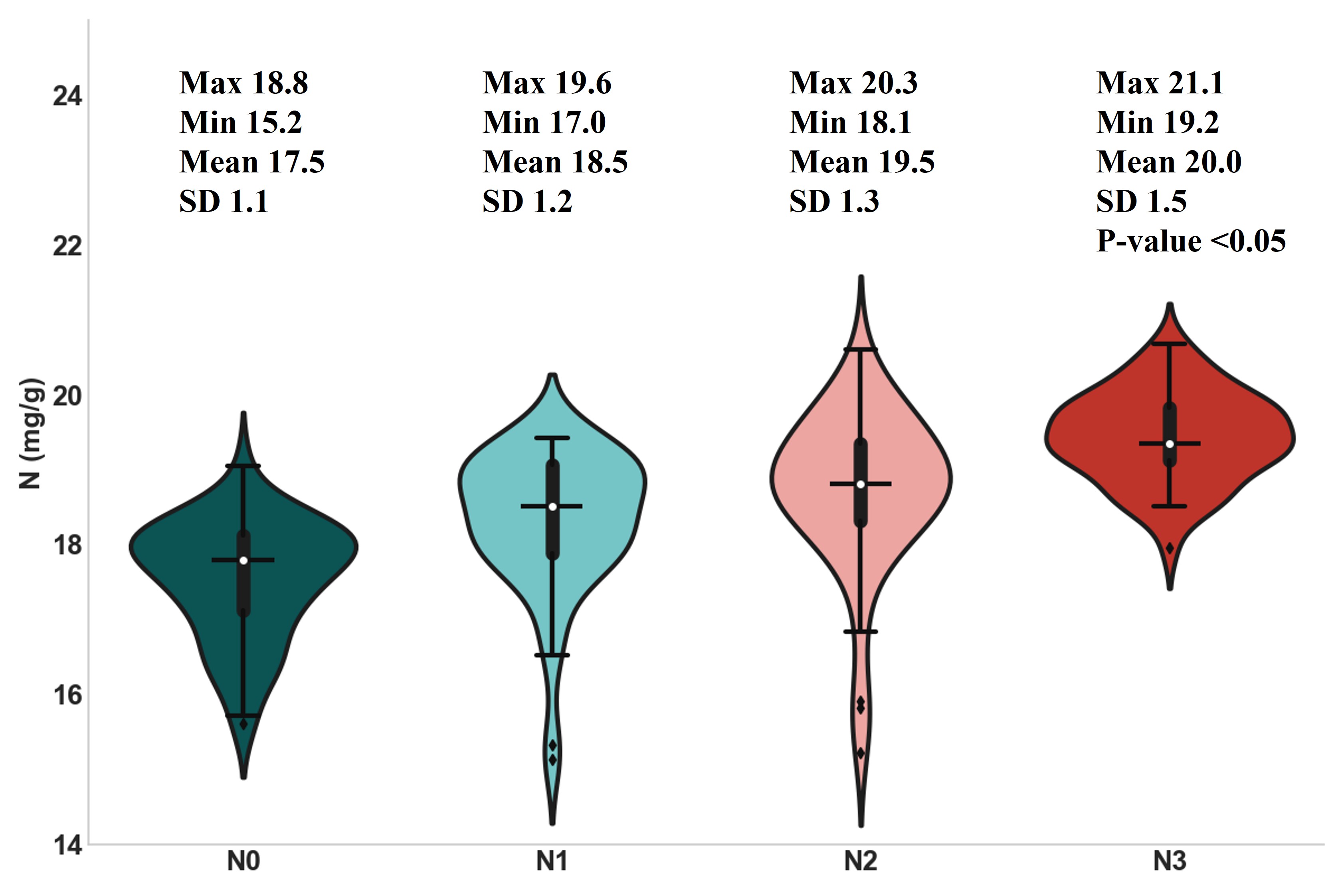


**Supplementary Figure 1.** Statistics of nitrogen content at different fertilization gradients


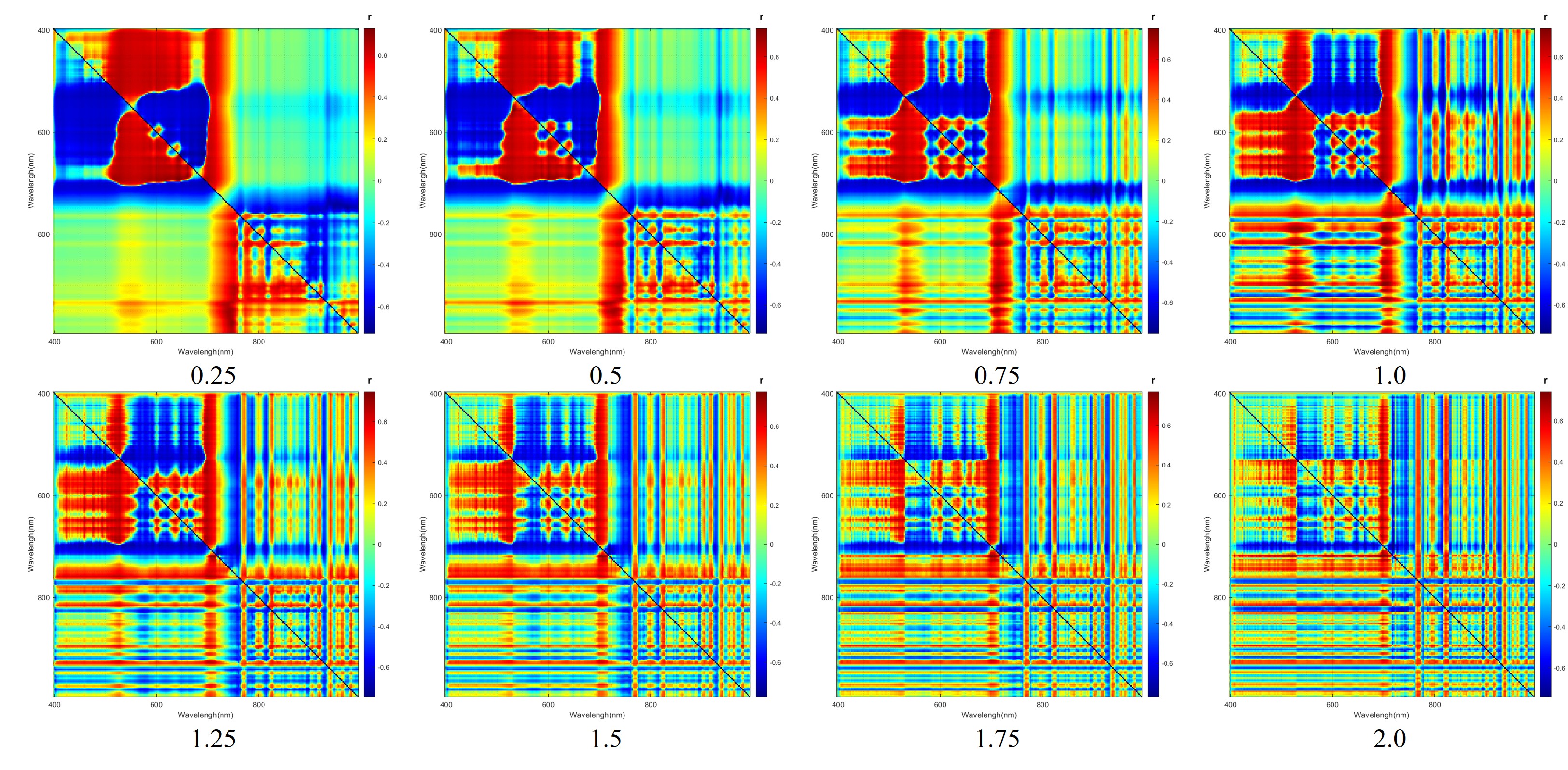


**Supplementary Figure 2.** Pearson correlation matrix between DI with different fractional orders and nitrogen content.


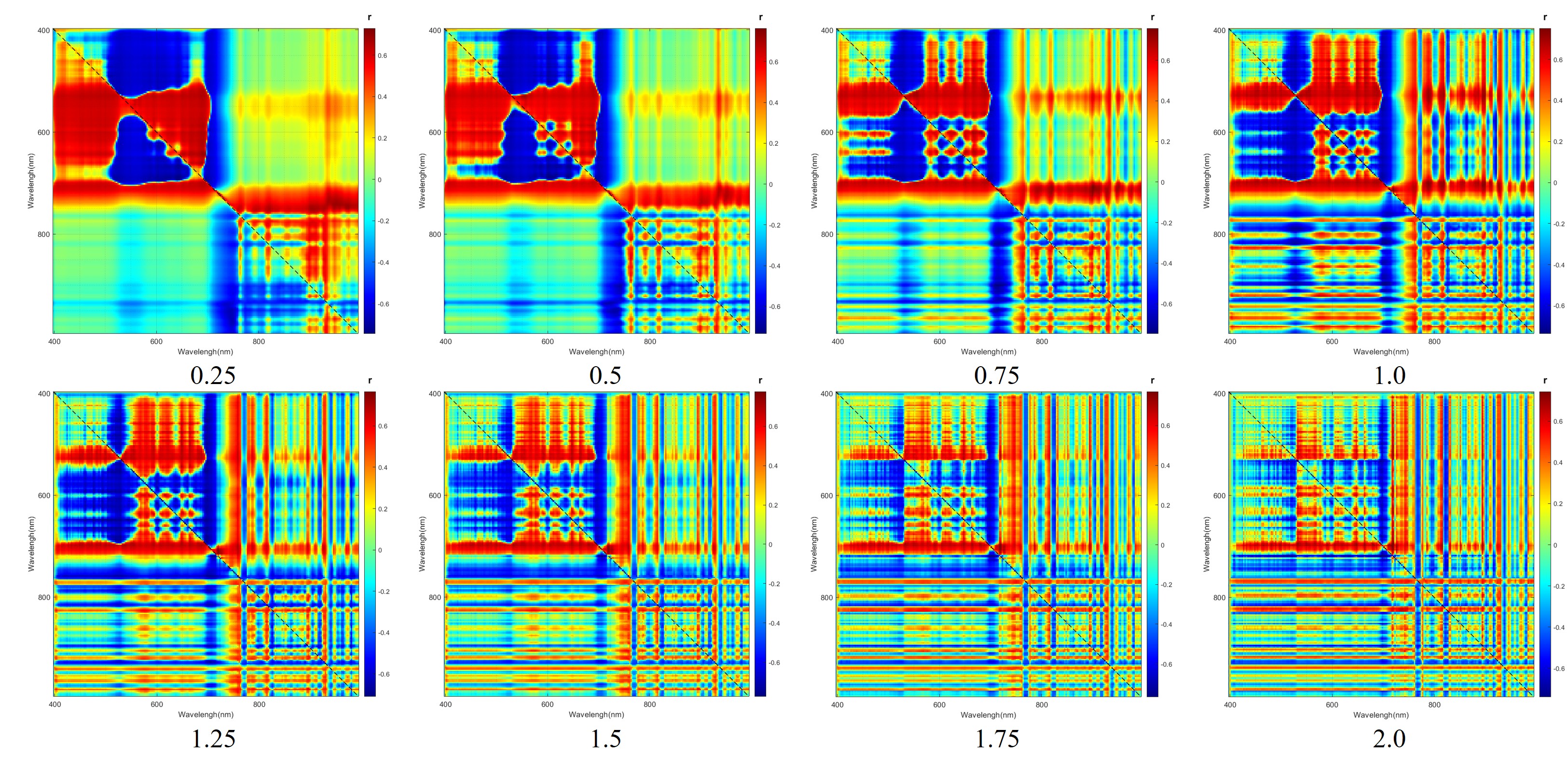


**Supplementary Figure 3.** Pearson correlation matrix between OSI with different fractional orders and nitrogen content.


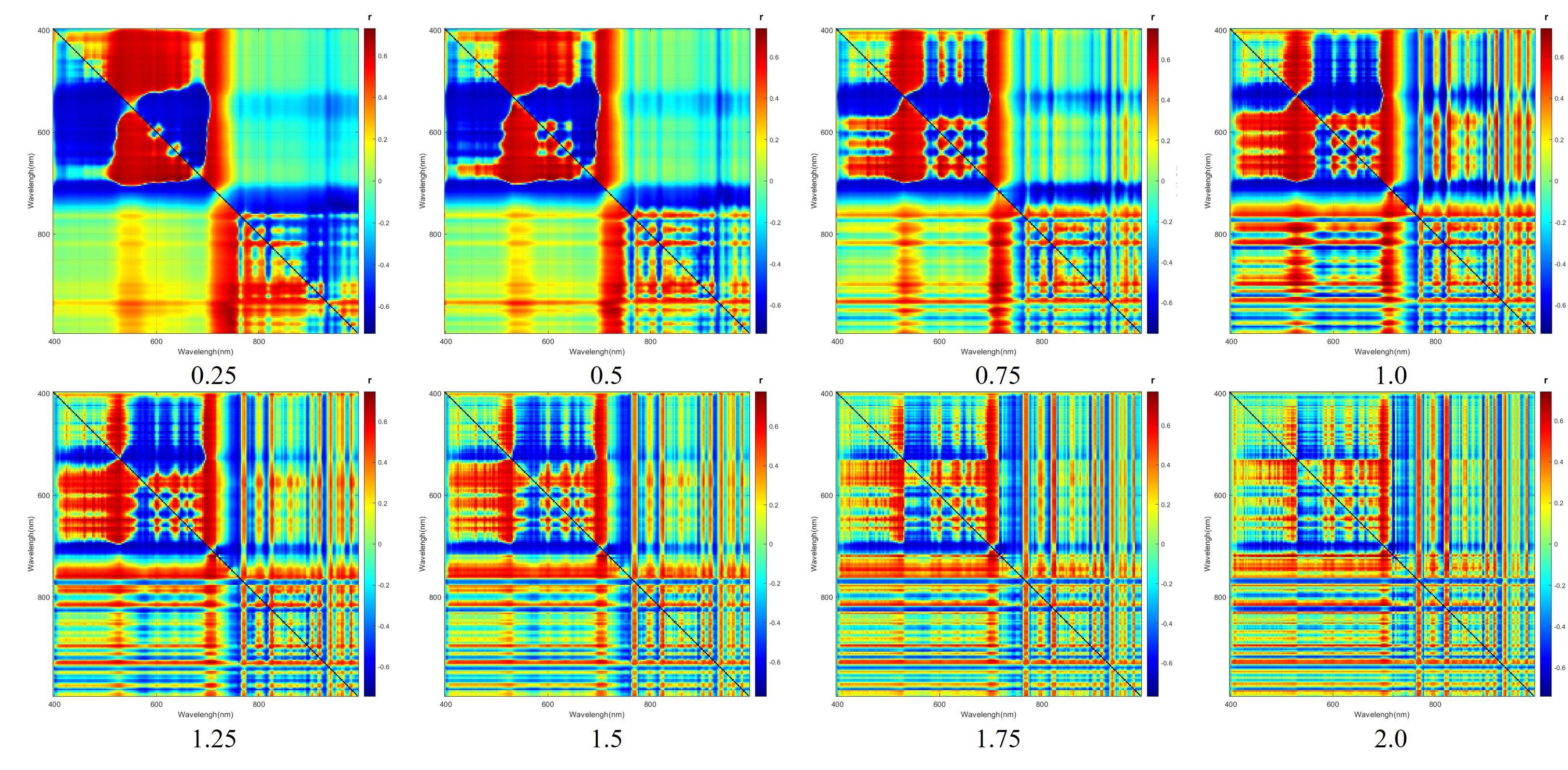


**Supplementary Figure 4.** Pearson correlation matrix between SASI with different fractional orders and nitrogen content.


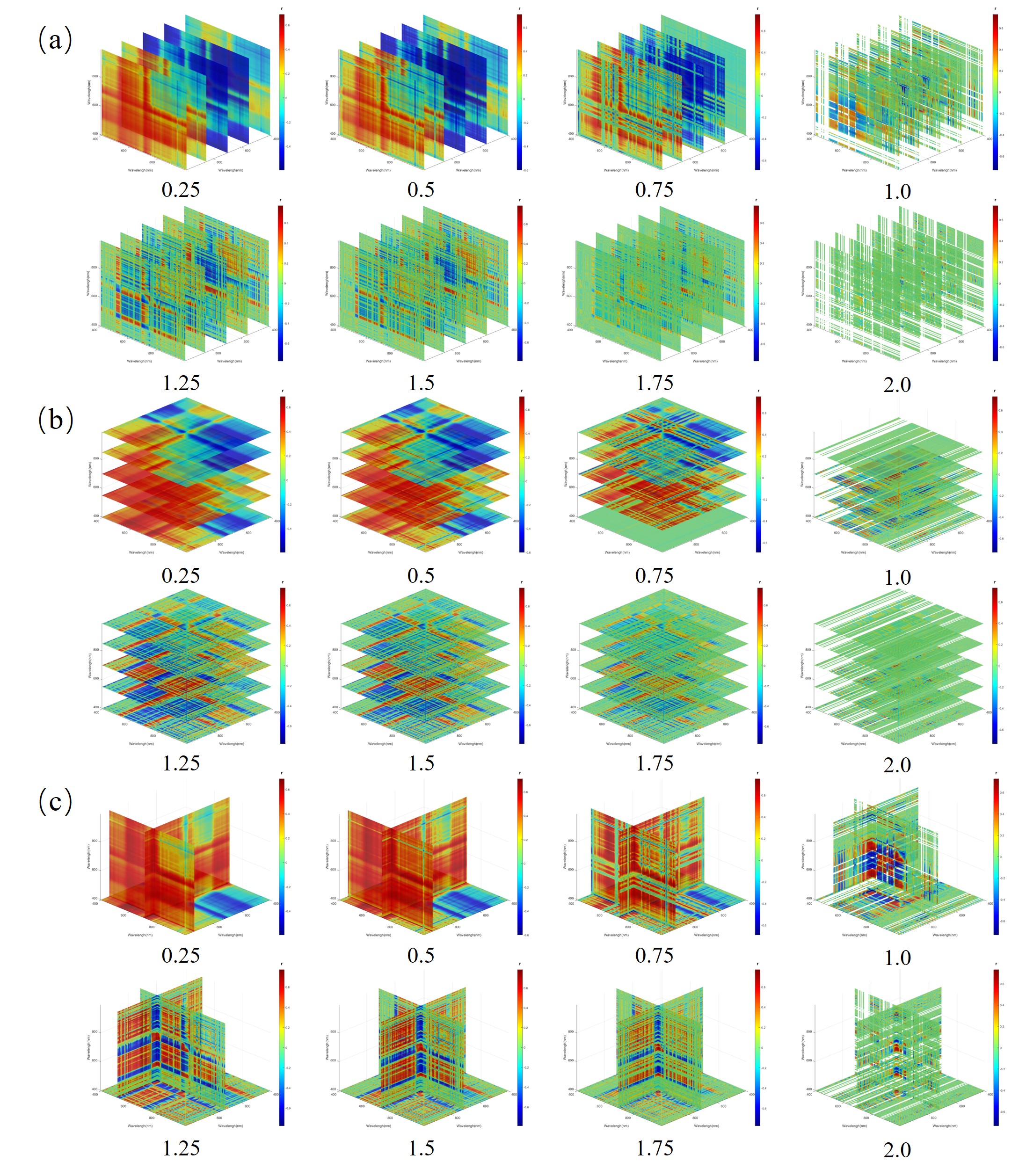


**Supplementary Figure 5.** Pearson correlation matrix between TBI1 with different fractional orders and nitrogen content.


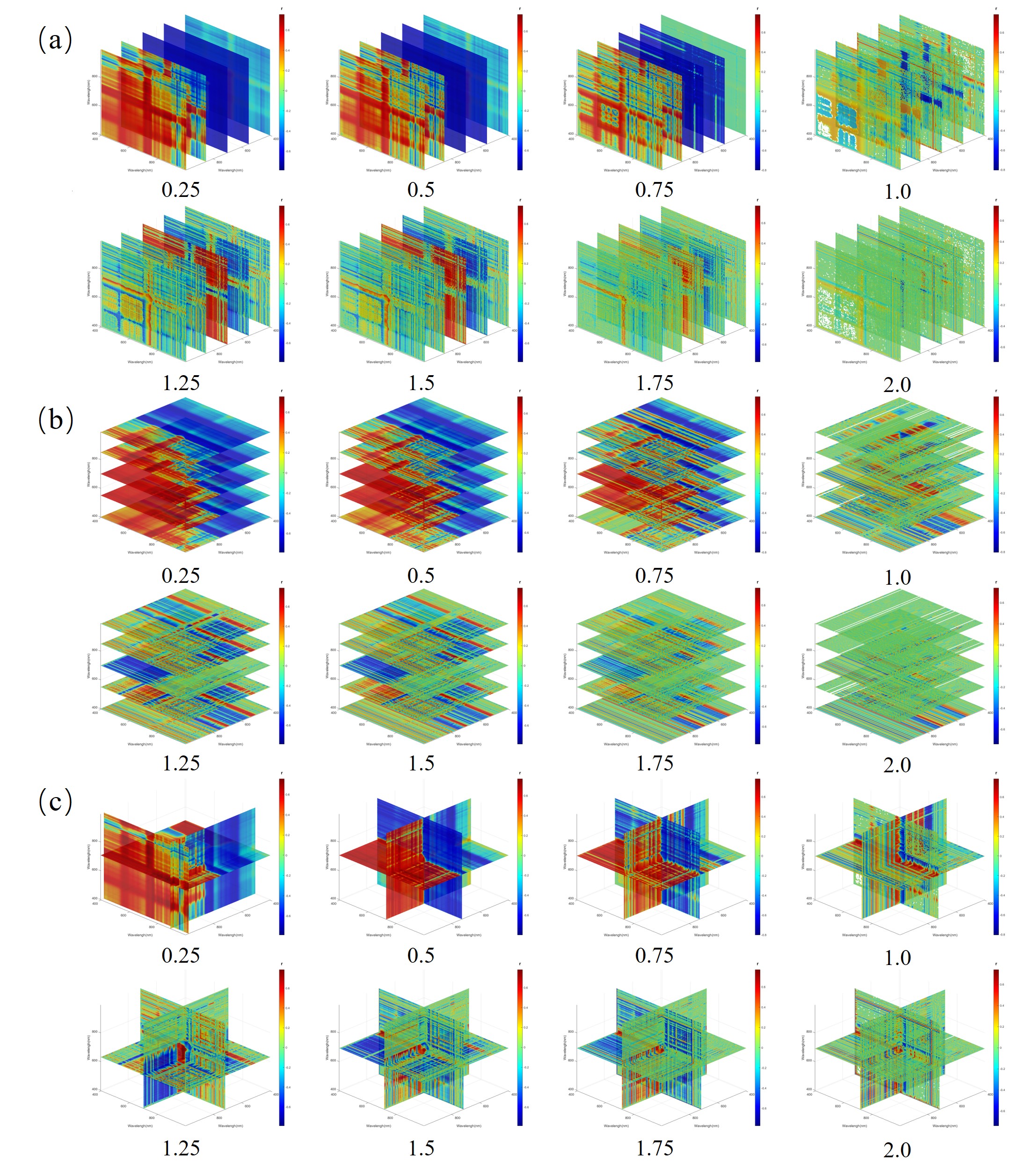


**Supplementary Figure 6.** Pearson correlation matrix between TBI2 with different fractional orders and nitrogen content.


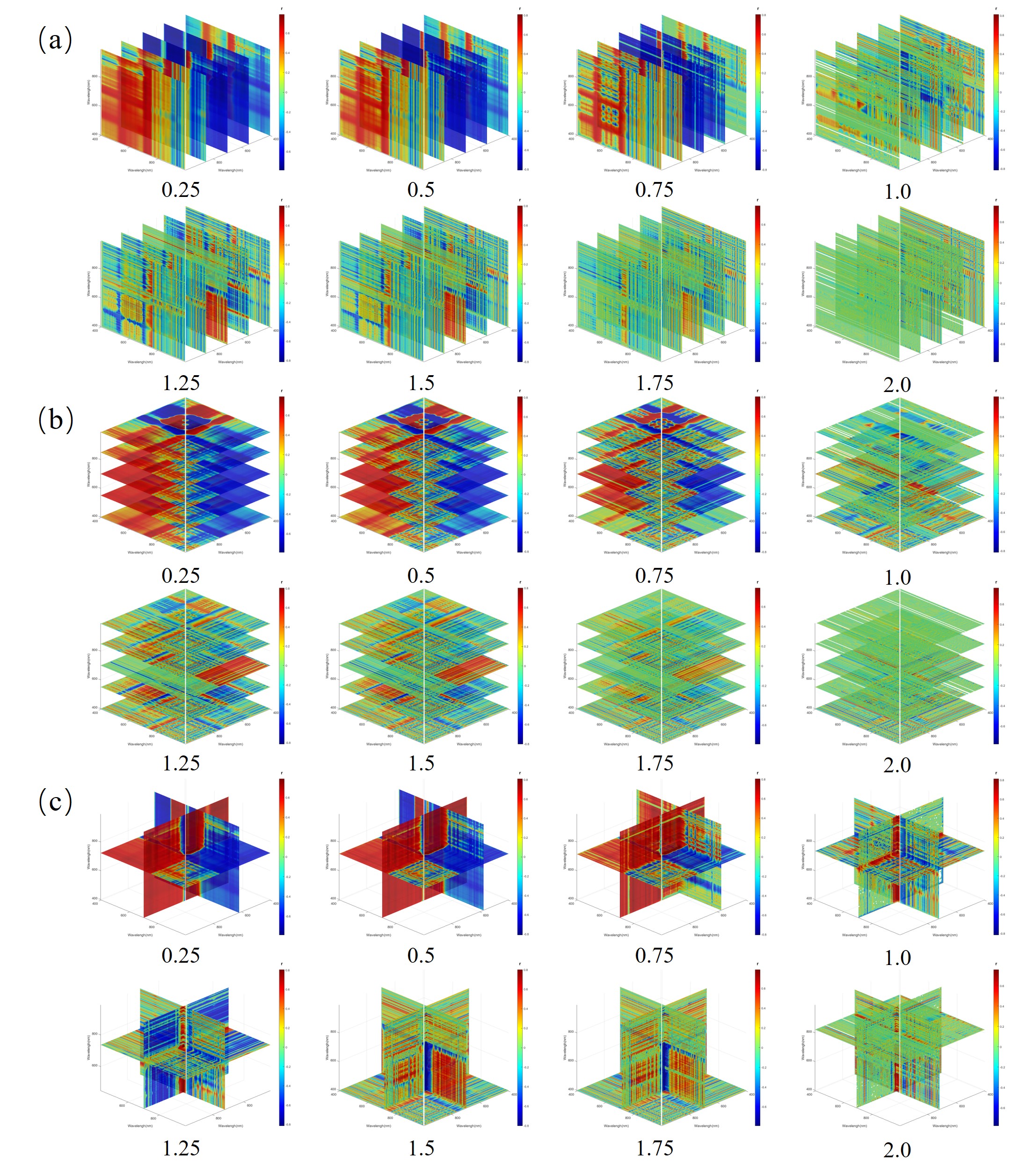


**Supplementary Figure 7.** Pearson correlation matrix between TBI3 with different fractional orders and nitrogen content.


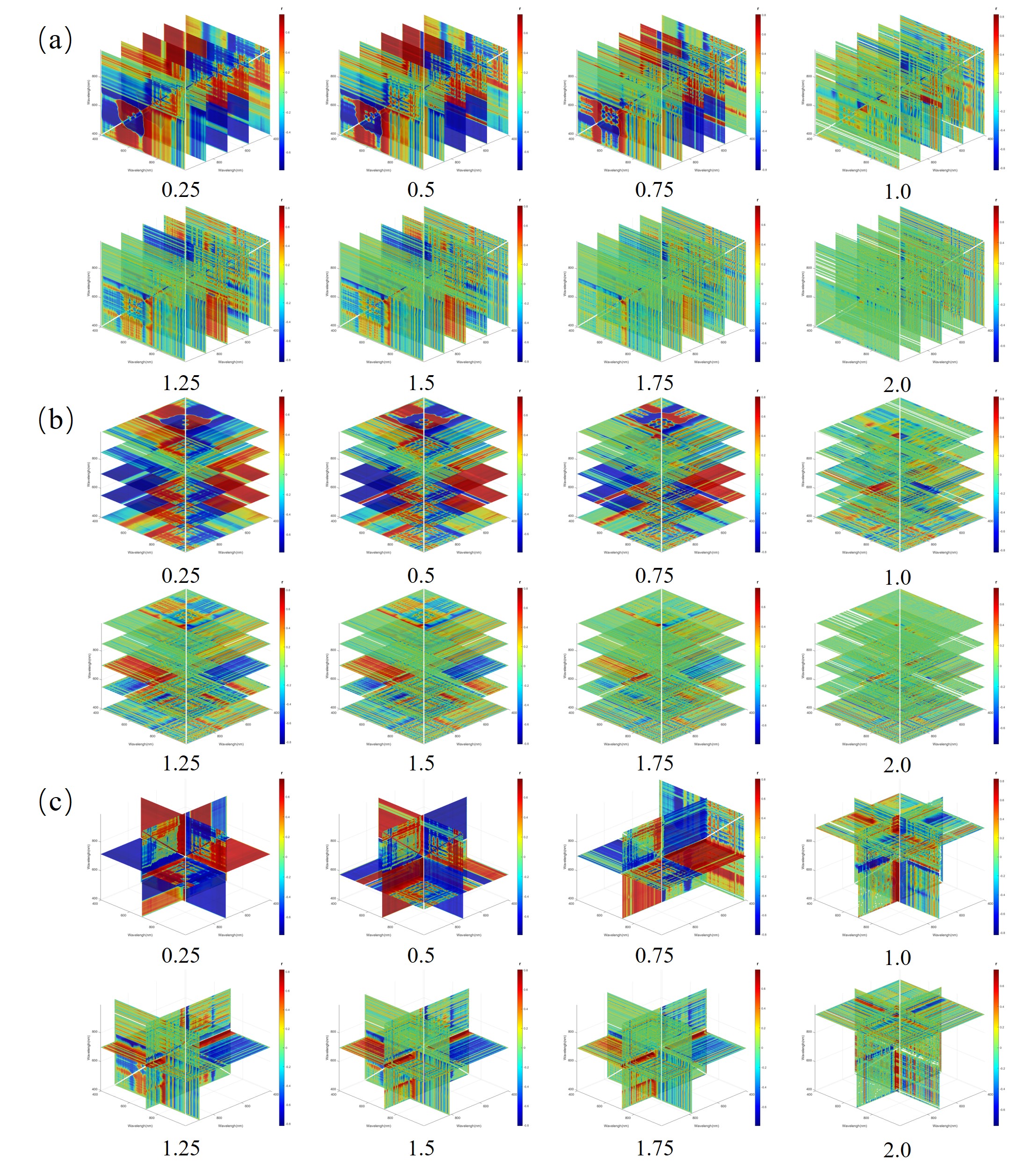


**Supplementary Figure 8.** Pearson correlation matrix between TBI4 with different fractional orders and nitrogen content.


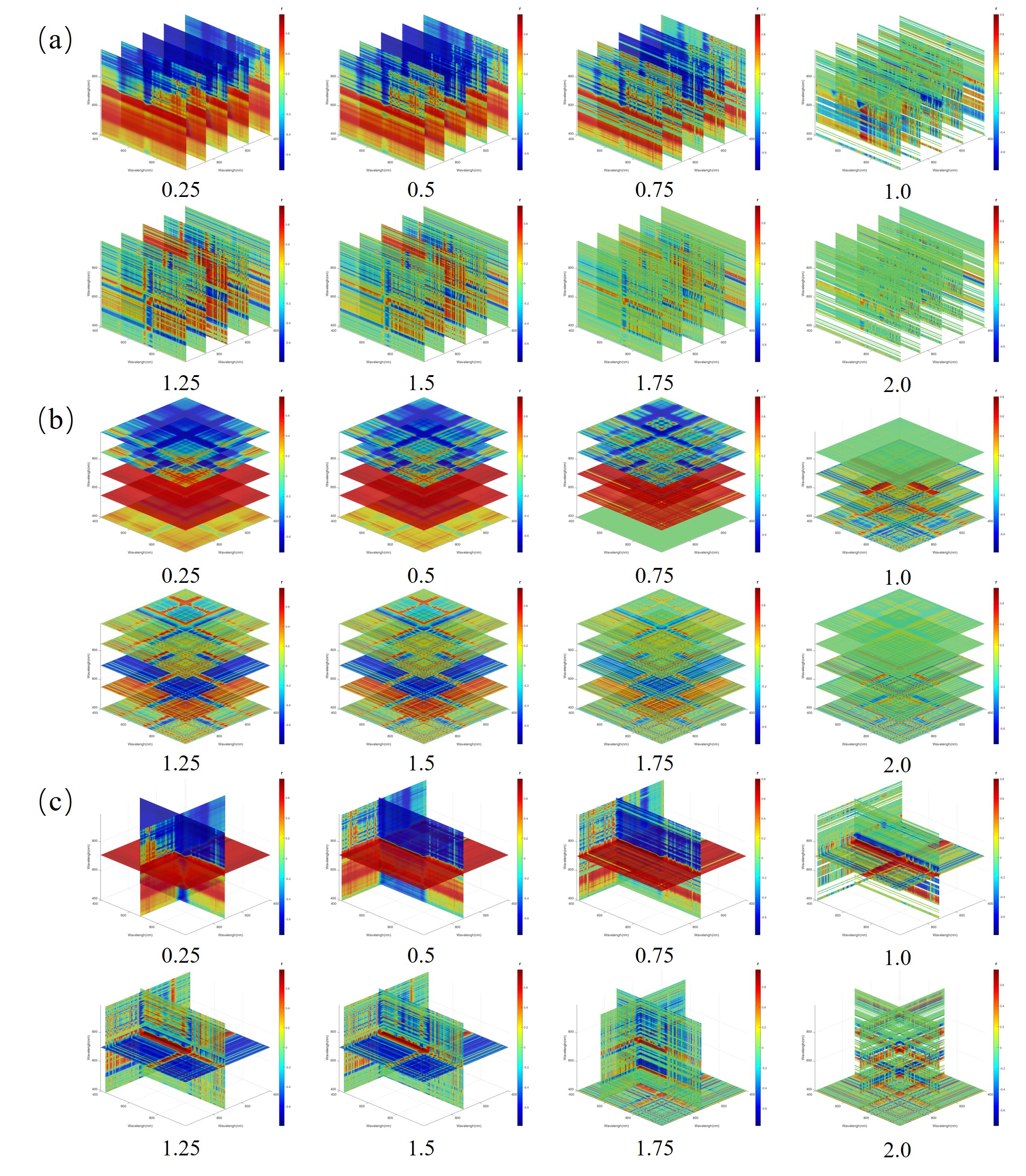


**Supplementary Figure 9.** Pearson correlation matrix between TBI5 with different fractional orders and nitrogen content.


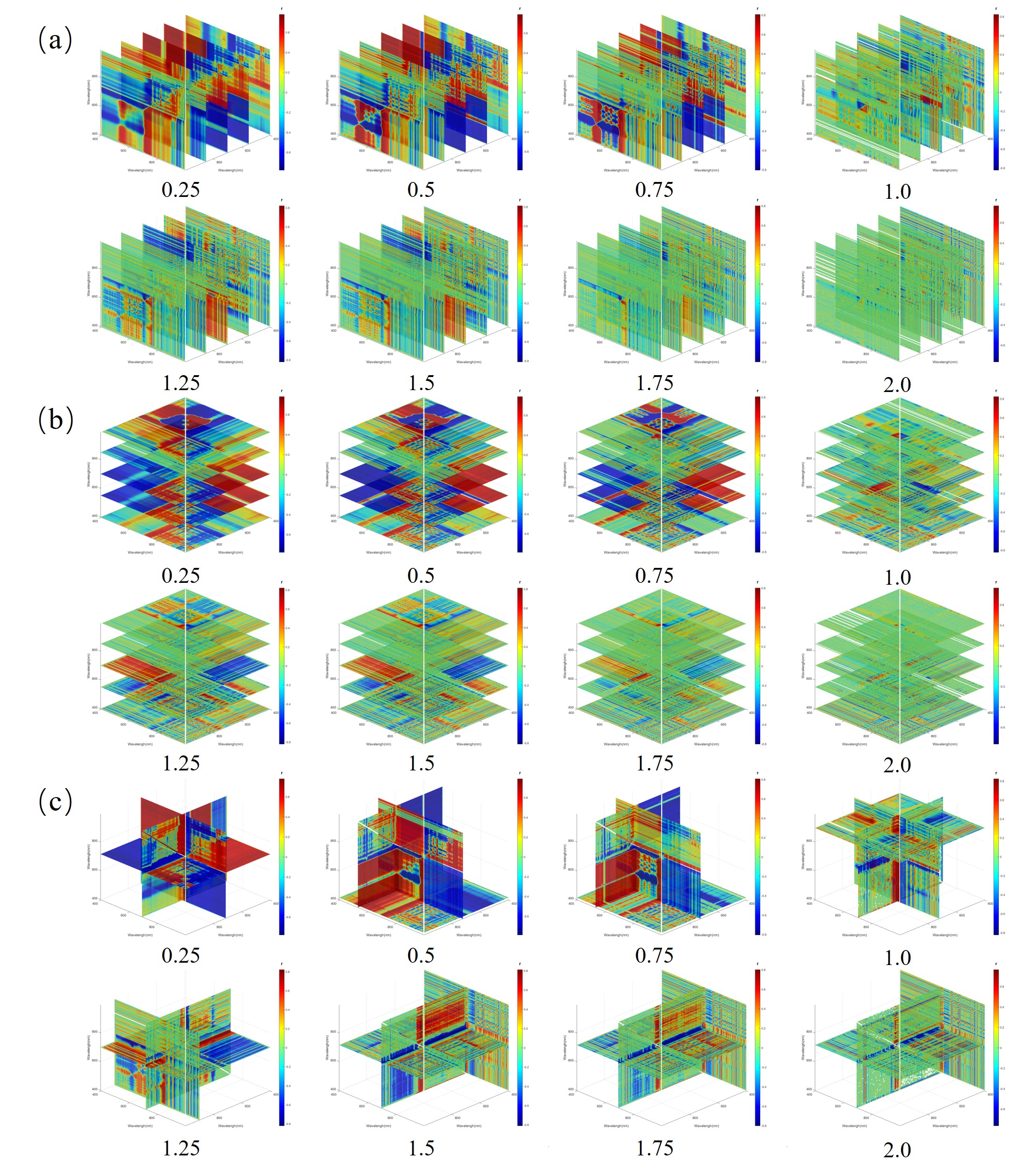


**Supplementary Figure 10.** Pearson correlation matrix between TBI6 with different fractional orders and nitrogen content.


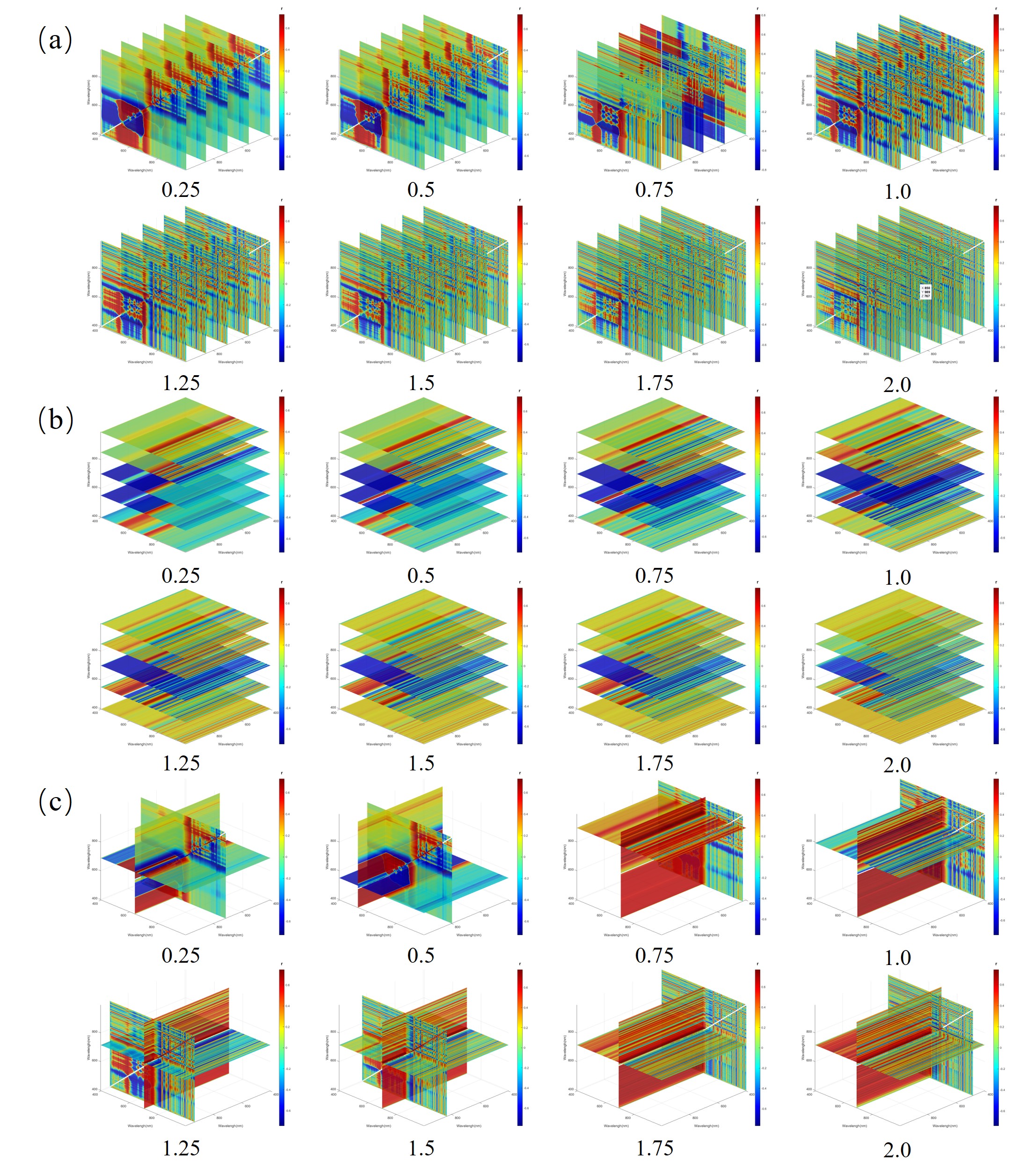
**Supplementary Figure 11.** Pearson correlation matrix between TBI7 with different fractional orders and nitrogen content.

## Supplementary Table

**Supplementary Table 1.** Main technical specifications of the GaiaSky-mini3 airborne hyperspectral imager

| Technical indicators | Technical Specifications |
| --- | --- |
| Spectral camera model | Gaiasky-mini3-VN |
| Spectral range | 400-1000（nm） |
| Spectral resolution | 5.5nm |
| Spectral sampling rate | 2.7nm@224 |
| Pixel size | 19.9×9.97（um） |
| Camera output | 12bit |
| Working voltage | 12v |
| Power | 45w |
| Image resolution | 1024×1024 |
| Number of spectral channels | 224 |
| Camera shot | 16mm |
| Spatial resolution | 0.062（@16mm,100m） |

**Supplementary Table 2.** Hyperparameter search ranges used in the grid search for the stacked ensemble model

| **Model** | **Hyperparameter** | **Range** |
| --- | --- | --- |
| XGBoost | n_estimators | 100–500 |
|  | learning_rate | 0.01–0.3 |
|  | max_depth | 1–10 |
|  | subsample | 0.6–1.0 |
|  | colsample_bytree | 0.6–1.0 |
|  | reg_alpha | 0-0.5 |
|  | reg_lambda | 0.5-2 |
| GBDT | n_estimators | 100–500 |
|  | learning_rate | 0.01–0.2 |
|  | max_depth | 1–10 |
|  | subsample | 0.6–1.0 |
|  | min_samples_split | 2-10 |
| Ridge | alpha | 0.01–10 |

**Supplementary Table 3.** The inversion results based on different models and different feature selection strategies

| Feature set | Model | Training sets | | Test sets | |
| --- | --- | --- | --- | --- | --- |
|  |  | R^2^c | RMSEC | R^2^p | RMSEP |
| RAW | PLSR | 0.805 | 0.484 | 0.726 | 0.565 |
|  | SVR | 0.774 | 0.521 | 0.733 | 0.558 |
|  | Stacking | 0.942 | 0.264 | 0.730 | 0.561 |
| GA | PLSR | 0.788 | 0.506 | 0.749 | 0.541 |
|  | SVR | 0.790 | 0.503 | 0.734 | 0.557 |
|  | Stacking | 0.926 | 0.298 | 0.735 | 0.556 |
| SPA | PLSR | 0.829 | 0.453 | 0.744 | 0.546 |
|  | SVR | 0.837 | 0.443 | 0.729 | 0.562 |
|  | Stacking | 0.912 | 0.325 | 0.737 | 0.554 |
| GA-SPA | PLSR | 0.755 | 0.543 | 0.701 | 0.590 |
|  | SVR | 0.825 | 0.458 | 0.746 | 0.544 |
|  | Stacking | 0.905 | 0.338 | 0.761 | 0.528 |

**Supplementary Table 4.** The maximum correlation coefficients between different orders of two-dimensional spectral indices and nitrogen content, as well as the optimal band combination

| FOD | DI | | OSI | | SASI | |
| --- | --- | --- | --- | --- | --- | --- |
|  | OBC | r | OBC | r | OBC | r |
| 0.25 | (R688,R639) | 0.728 | (R712,R715) | 0.730 | (R688,R639) | 0.731 |
| 0.5 | (R550,R529) | 0.739 | (R712,R715) | 0.764 | (R550,R529) | 0.738 |
| 0.75 | (R897,R704) | 0.754 | (R707,R897) | 0.757 | (R897,R707) | 0.754 |
| 1.0 | (R783,R696) | 0.739 | (R709,R715) | 0.752 | (R783,R696) | 0.739 |
| 1.25 | (R715,R704) | 0.746 | (R701,R715) | 0.766 | (R715,R704) | 0.747 |
| 1.5 | (R715,R701) | 0.778 | (R701,R715) | 0.776 | (R715,R701) | 0.778 |
| 1.75 | (R715,R693) | 0.770 | (R693,R715) | 0.771 | (R715,R693) | 0.770 |
| 2.0 | (R715,R690) | 0.743 | (R690,R715) | 0.744 | (R715,R690) | 0.743 |

**Supplementary Table 5.** The maximum correlation coefficients between different orders of three-dimensional spectral indices and nitrogen content, as well as the optimal band combination

| FOD |  | TBI1 | TBI2 | TBI3 | TBI4 | TBI5 | TBI6 | TBI7 |
| --- | --- | --- | --- | --- | --- | --- | --- | --- |
| 0.25 | OBC | (R933,R707,R397) | (R972,R888,R707) | (R617,R701,R723) | (R712,R688,R715) | (R718,R677,R709) | (R712,R688,R715) | (R712,R639,R688) |
|  | r | 0.688 | 0.756 | 0.790 | 0.781 | 0.788 | 0.781 | 0.728 |
| 0.5 | OBC | (R933,R701,R397) | (R726,R729,R707) | (R574,R701,R718) | (R718,R701,R574) | (R715,R415,R709) | (R720,R529,R413) | (R797,R529,R550) |
|  | r | 0.712 | 0.769 | 0.810 | 0.788 | 0.798 | 0.790 | 0.739 |
| 0.75 | OBC | (R897,R699,R397) | (R726,R729,R704) | (R574,R701,R715) | (R413,R718,R701) | (R718,R413,R701) | (R720,R529,R413) | (R421,R707,R897) |
|  | r | 0.753 | 0.782 | 0.806 | 0.806 | 0.803 | 0.807 | 0.754 |
| 1.0 | OBC | (R720,R526,R397) | (R723,R726,R701) | (R720,R704,R737) | (R718,R701,R897) | (R718,R413,R701) | (R718,R701,R897) | (R397,R696,R783) |
|  | r | 0.729 | 0.786 | 0.813 | 0.823 | 0.801 | 0.824 | 0.739 |
| 1.25 | OBC | (R715,R516,R397) | (R715,R699,R631) | (R720,R701,R737) | (R897,R715,R699) | (R715,R431,R699) | (R897,R715,R699) | (R930,R704,R715) |
|  | r | 0.774 | 0.788 | 0.805 | 0.823 | 0.791 | 0.822 | 0.746 |
| 1.5 | OBC | (R715,R693,R397) | (R715,R715,R693) | (R696,R715,R400) | (R814,R715,R696) | (R715,R431,R699) | (R397,R696,R715) | (R833,R701,R715) |
|  | r | 0.785 | 0.781 | 0.801 | 0.806 | 0.779 | 0.811 | 0.778 |
| 1.75 | OBC | (R715,R690,R397) | (R715,R715,R693) | (R690,R715,R400) | (R781,R715,R693) | (R715,R564,R397) | (R397,R690,R715) | (R397,R693,R715) |
|  | r | 0.772 | 0.772 | 0.791 | 0.792 | 0.771 | 0.803 | 0.770 |
| 2.0 | OBC | (R715,R690,R397) | (R715,R715,R690) | (R715,R690,R822) | (R715,R690,R927) | (R715,R712,R397) | (R397,R690,R715) | (R481,R690,R715) |
|  | r | 0.733 | 0.741 | 0.760 | 0.759 | 0.748 | 0.765 | 0.743 |

**Supplementary Table 6.** Based on the inversion results of different models and different spectral indices

| Feature set | Model | Training sets | | Test sets | |
| --- | --- | --- | --- | --- | --- |
|  |  | R^2^c | RMSEC | R^2^p | RMSEP |
| 2-SIs | PLSR | 0.714 | 0.587 | 0.719 | 0.573 |
|  | SVR | 0.719 | 0.581 | 0.724 | 0.567 |
|  | Stacking | 0.720 | 0.581 | 0.714 | 0.577 |
| 3-SIs | PLSR | 0.797 | 0.484 | 0.785 | 0.500 |
|  | SVR | 0.796 | 0.495 | 0.772 | 0.516 |
|  | Stacking | 0.910 | 0.329 | 0.801 | 0.481 |
| SIs | PLSR | 0.799 | 0.492 | 0.790 | 0.494 |
|  | SVR | 0.797 | 0.494 | 0.770 | 0.518 |
|  | Stacking | 0.906 | 0.336 | 0.805 | 0.477 |

**Supplementary Table 7.** Inversion results based on different models and the fusion of different features

| Feature set | Model | Training sets | | Test sets | |
| --- | --- | --- | --- | --- | --- |
|  |  | R^2^c | RMSEC | R^2^p | RMSEP |
| Spectra | PLSR | 0.755 | 0.543 | 0.701 | 0.590 |
|  | SVR | 0.825 | 0.458 | 0.746 | 0.544 |
|  | RF | 0.844 | 0.433 | 0.606 | 0.677 |
|  | GBDT | 0.932 | 0.286 | 0.669 | 0.621 |
|  | XGB | 0.977 | 0.166 | 0.715 | 0.576 |
|  | Stacking | 0.905 | 0.338 | 0.761 | 0.528 |
| SIs | PLSR | 0.799 | 0.492 | 0.790 | 0.494 |
|  | SVR | 0.797 | 0.494 | 0.770 | 0.518 |
|  | RF | 0.857 | 0.415 | 0.782 | 0.504 |
|  | GBDT | 0.906 | 0.337 | 0.783 | 0.503 |
|  | XGB | 0.881 | 0.379 | 0.780 | 0.506 |
|  | Stacking | 0.906 | 0.336 | 0.805 | 0.477 |
| VIs | PLSR | 0.498 | 0.778 | 0.279 | 0.917 |
|  | SVR | 0.508 | 0.770 | 0.225 | 0.950 |
|  | RF | 0.504 | 0.773 | 0.115 | 1.015 |
|  | GBDT | 0.704 | 0.597 | 0.078 | 1.037 |
|  | XGB | 0.578 | 0.713 | 0.122 | 1.012 |
|  | Stacking | 0.619 | 0.677 | 0.209 | 0.960 |
| Spectra-VIs | PLSR | 0.799 | 0.492 | 0.749 | 0.541 |
|  | SVR | 0.762 | 0.535 | 0.750 | 0.539 |
|  | RF | 0.797 | 0.494 | 0.544 | 0.729 |
|  | GBDT | 0.939 | 0.272 | 0.622 | 0.663 |
|  | XGB | 0.931 | 0.289 | 0.626 | 0.660 |
|  | Stacking | 0.901 | 0.345 | 0.766 | 0.523 |
| Spectra-SIs | PLSR | 0.845 | 0.432 | 0.782 | 0.504 |
|  | SVR | 0.850 | 0.425 | 0.797 | 0.486 |
|  | RF | 0.930 | 0.291 | 0.791 | 0.494 |
|  | GBDT | 0.972 | 0.183 | 0.774 | 0.513 |
|  | XGB | 0.905 | 0.338 | 0.793 | 0.491 |
|  | Stacking | 0.927 | 0.297 | 0.815 | 0.464 |
| Spectra-Vis-SIs | PLSR | 0.849 | 0.427 | 0.798 | 0.485 |
|  | SVR | 0.823 | 0.462 | 0.804 | 0.477 |
|  | RF | 0.903 | 0.341 | 0.782 | 0.504 |
|  | GBDT | 0.927 | 0.297 | 0.794 | 0.490 |
|  | XGB | 0.916 | 0.318 | 0.791 | 0.494 |
|  | Stacking | 0.918 | 0.314 | 0.826 | 0.450 |
